# The use of machine learning on administrative and survey data to predict suicidal thoughts and behaviors: a systematic review

**DOI:** 10.3389/fpsyt.2024.1291362

**Published:** 2024-03-04

**Authors:** Nibene H. Somé, Pardis Noormohammadpour, Shannon Lange

**Affiliations:** ^1^ Institute for Mental Health Policy Research, Centre for Addiction and Mental Health, Toronto, ON, Canada; ^2^ Campbell Family Mental Health Research Institute, Centre for Addiction and Mental Health, Toronto, ON, Canada; ^3^ Department of Epidemiology and Biostatistics, Schulich School of Medicine & Dentistry, Western University, London, ON, Canada; ^4^ Dalla Lana School of Public Health, University of Toronto, Toronto, ON, Canada; ^5^ Department of Psychiatry, University of Toronto, Toronto, ON, Canada

**Keywords:** death by suicide, suicidal thoughts, suicide attempt, machine learning, predictive risk factors

## Abstract

**Background:**

Machine learning is a promising tool in the area of suicide prevention due to its ability to combine the effects of multiple risk factors and complex interactions. The power of machine learning has led to an influx of studies on suicide prediction, as well as a few recent reviews. Our study distinguished between data sources and reported the most important predictors of suicide outcomes identified in the literature.

**Objective:**

Our study aimed to identify studies that applied machine learning techniques to administrative and survey data, summarize performance metrics reported in those studies, and enumerate the important risk factors of suicidal thoughts and behaviors identified.

**Methods:**

A systematic literature search of PubMed, Medline, Embase, PsycINFO, Web of Science, Cumulative Index to Nursing and Allied Health Literature (CINAHL), and Allied and Complementary Medicine Database (AMED) to identify all studies that have used machine learning to predict suicidal thoughts and behaviors using administrative and survey data was performed. The search was conducted for articles published between January 1, 2019 and May 11, 2022. In addition, all articles identified in three recently published systematic reviews (the last of which included studies up until January 1, 2019) were retained if they met our inclusion criteria. The predictive power of machine learning methods in predicting suicidal thoughts and behaviors was explored using box plots to summarize the distribution of the area under the receiver operating characteristic curve (AUC) values by machine learning method and suicide outcome (i.e., suicidal thoughts, suicide attempt, and death by suicide). Mean AUCs with 95% confidence intervals (CIs) were computed for each suicide outcome by study design, data source, total sample size, sample size of cases, and machine learning methods employed. The most important risk factors were listed.

**Results:**

The search strategy identified 2,200 unique records, of which 104 articles met the inclusion criteria. Machine learning algorithms achieved good prediction of suicidal thoughts and behaviors (i.e., an AUC between 0.80 and 0.89); however, their predictive power appears to differ across suicide outcomes. The boosting algorithms achieved good prediction of suicidal thoughts, death by suicide, and all suicide outcomes combined, while neural network algorithms achieved good prediction of suicide attempts. The risk factors for suicidal thoughts and behaviors differed depending on the data source and the population under study.

**Conclusion:**

The predictive utility of machine learning for suicidal thoughts and behaviors largely depends on the approach used. The findings of the current review should prove helpful in preparing future machine learning models using administrative and survey data.

**Systematic review registration:**

https://www.crd.york.ac.uk/prospero/display_record.php?ID=CRD42022333454 identifier CRD42022333454.

## Introduction

Suicide is a significant public health problem. In 2019, approximately 703,000 people died by suicide worldwide, for a global age-standardized suicide mortality rate of 9.0 per 100,000 population (1). Given this burden, international goals to reduce the suicide mortality rate have been set. Prevention is critical to achieving such goals (2, 3). In 2021, the World Health Organization released the *LIVE LIFE: an implementation guide for suicide prevention in countries*, describing four effective evidence-based interventions to prevent suicide (4). These include 1) limiting access to the means of suicide, 2) interacting with the media for responsible reporting of suicide, 3) fostering socio-emotional life skills in adolescents, and 4) early identification, assessment, management, and follow-up of anyone at-risk of suicidal behavior. With respect to the latter, identifying those at-risk of suicidal behaviors (i.e., a target population) is critical. While it has been suggested that history of suicidal ideation and past suicide attempts could help predict future suicidal behavior (5), it is evident that many more factors are involved, which makes traditional prediction approaches inefficient (6).

While conventional prediction approaches apply statistical models with a limited number of predictors, mediators, and interactions, in the last decade, machine learning has become a valuable tool with a lot of promise in the area of suicide prevention due to its ability to combine the effects of multiple risk factors and complex interactions (7). In addition, conventional prediction techniques depend on the researcher’s definition of the relationship between predictors and outcomes. However, machine learning approaches can examine all potential relationships repetitively and detect the most accurate prediction algorithm (7).

Given the power of machine learning for prediction purposes, many studies have applied this technique to identify risk factors that are predictive of suicidal thoughts and behaviors in recent years. Thus, to extract applicable findings, existing systematic reviews have investigated the extent to which these machine learning techniques have been used and assessed the predictive validity of published models (8–10). However, the existing systematic reviews did not differentiate between data sources and have largely failed to summarize the most important risk factors listed in the studies.

Considering the accessibility of administrative and survey data, the current study aimed to identify all existing studies that applied machine learning techniques to such data to predict suicidal thoughts and behaviors. The objective was two-fold, to: i) summarize performance metrics reported in the studies by study design, data source, sample size and type of machine learning methods; and ii) enumerate predictors identified in the studies as important contributors to the model performance at predicting suicidal thoughts and behaviors by study design, data source, and suicide outcomes.

## Methods

The current systematic review is reported according to the Preferred Reporting Items for Systematic Reviews and Meta-Analyses (PRISMA) 2020 statement (11). The protocol was registered with the International Prospective Register of Systematic Reviews (PROSPERO, registration number CRD42022333454).

### Outcome definitions

The following outcomes were of interest: suicidal ideation, suicide plan, suicide attempts, and death by suicide; the definitions of which are in line with those of the Center for Disease Control and Prevention (12). The term suicidal thoughts is used herein to capture suicidal ideation and suicide plans. Suicidal ideation was defined as thoughts of engaging in suicide-related behavior; and suicide plan was defined as a thought regarding a self-initiated action that facilitates self-harm behavior or a suicide attempt; this will often include an organized manner of engaging in suicidal behavior, such as a description of a time frame and method. Suicide attempt was defined as a non-fatal self-directed potentially injurious behavior with any intent to die as a result of the behavior, and death by suicide was defined as death caused by self-directed injurious behavior with any intent to die as a result of the behavior.

### Search strategy and selection criteria

A systematic literature search of PubMed, Medline, Embase, PsycINFO, Web of Science, Cumulative Index to Nursing and Allied Health Literature (CINAHL), and Allied and Complementary Medicine Database (AMED) was performed to identify all studies that have used machine learning to predict suicidal thoughts and behaviors with administrative or survey data (keywords are presented in **Appendix 1**). The searches were not limited geographically or by language of publication. Considering that three recent systematic reviews (8–10) have been published in this field, we conducted our search for articles published between January 1, 2019 and May 11, 2022. We retained all articles identified in the respective systematic reviews, if they met our inclusion criteria. The citations in all included articles were also manually screened. To ascertain our findings, we conducted an independent search (i.e., ignoring the results of the three systematic reviews) using the same keywords and including published studies through May 11, 2022.

### Inclusion and exclusion criteria

Articles were included if they i) consisted of original, quantitative research published in a peer-reviewed journal or scholarly report; ii) used a machine learning method to predict a suicide outcome (suicidal ideation, suicide plan, suicide attempt, and/or death by suicide); and iii) used either an administrative or survey dataset containing individual-level data.

Articles were excluded if they performed a machine learning technique (e.g., natural language processing) to scan social media platforms to detect suicidal thoughts and behaviors. There was no restriction on population or study setting.

### Study selection and data extraction

Two investigators performed title and abstract screening and full-text reviews independently. Conflicts in study identification were resolved in conjunction with the third investigator. All screening was completed using EndNote 20, and data extraction was completed by one investigator using a template created in Microsoft Excel and cross-checked by the other two investigators. The following variables were extracted: country, study design, study duration, data source, study population, sample size, number of cases and controls, suicidal outcome, validation technique, machine learning methods, relevant risk factors (or most important predictive risk factors), and performance statistics, including accuracy, sensitivity, specificity, positive predictive value, negative predictive value, and area under the receiver operating characteristic curve (AUC).

### Risk of bias

Two investigators assessed the risk of bias using the Prediction model study Risk of Bias Assessment Tool (PROBAST) (13). In the presence of conflicts in evaluating the risk of bias in a study, the third investigator was consulted, and the discrepancies were discussed until a unanimous decision was taken. PROBAST contains twenty signaling questions in four domains: participants, predictors, outcome, and analysis. It also investigates the applicability of studies in participants, predictors, and outcome domains. Based on PROBAST, included studies were classified into one of three categories: low, high, and unclear risk of bias and applicability.

### Statistical analysis

Our statistical analysis aims to summarize performance metrics reported in the included studies. Meta-analysis is beyond the scope here; more details can be found in (14, 15). Our descriptive analysis includes two different steps. First, we used a box plot of the most frequently reported performance statistics (i.e., the AUC) to explore the predictive power of machine learning methods in predicting suicidal thoughts and behaviors. Box plots were used to summarize the distribution of the AUC values by suicide outcome and machine learning method. Additionally, box plots helped identify AUC values that differed significantly from the rest of the AUCs (i.e., outliers). The presence of these outliers may be due to the fact that we did not consider only the AUCs for the best model – all AUCs in the selected studies were reported. Note that an AUC of 0.5 indicates that the machine learning method performance is no better than chance (i.e., random guesses). AUCs above 0.9 indicate excellent prediction, between 0.80 and 0.89 good prediction, 0.70 to 0.79 fair prediction, 0.60 to 0.69 poor prediction and 0.50 to 0.59 extremely poor prediction (16). AUC interpretation can vary widely depending on studies objectives and the trade-off between false positives and false negatives. For example, suppose the objective was to minimize the false negatives in suicidal thoughts and behaviors. In that case, one might lower the above thresholds so that more positive events are classified as positive. However, accounting for the specific context of each included study is above the scope of this review as the information was not reported in the included articles. We then considered the thresholds abovementioned to assess the predictive power of each model and compare the models. Second, we computed mean AUCs for all the models and the mean AUCs for the best-performing models (i.e., with the higher AUCs) in each article with 95% confidence intervals (CIs) by suicide outcomes (i.e., suicidal thoughts, suicide attempt, and death by suicide, as well as all suicide outcomes combined) across different study subgroups and machine learning methods. We regrouped the studies by study design (cross-sectional and longitudinal), study data source (administrative, survey, or both), total sample size (≤1,000, 1,000 to 10,000 and >10,000), and sample size of cases (≤200, 200 to 1,000, and >1,000). We also regrouped the AUCs by machine learning methods employed, namely Bayesian algorithms (naïve Bayes, Bayesian network, or Bayesian additive regression trees), boosting algorithms [gradient boosting tree, adaptive boosting (AdaBoost), or extreme gradient boosting (XGBoost)], cox regression, decision tree, K-nearest neighbors, linear discriminant analysis, logistic regression, neural network, random forest, regularized regressions (elastic net, least absolute shrinkage and selection operator (LASSO) or ridge regression), super learner (i.e., a combination of machine learning algorithms), and support vector machine.

## Results

### Study selection

**Figure 1:**
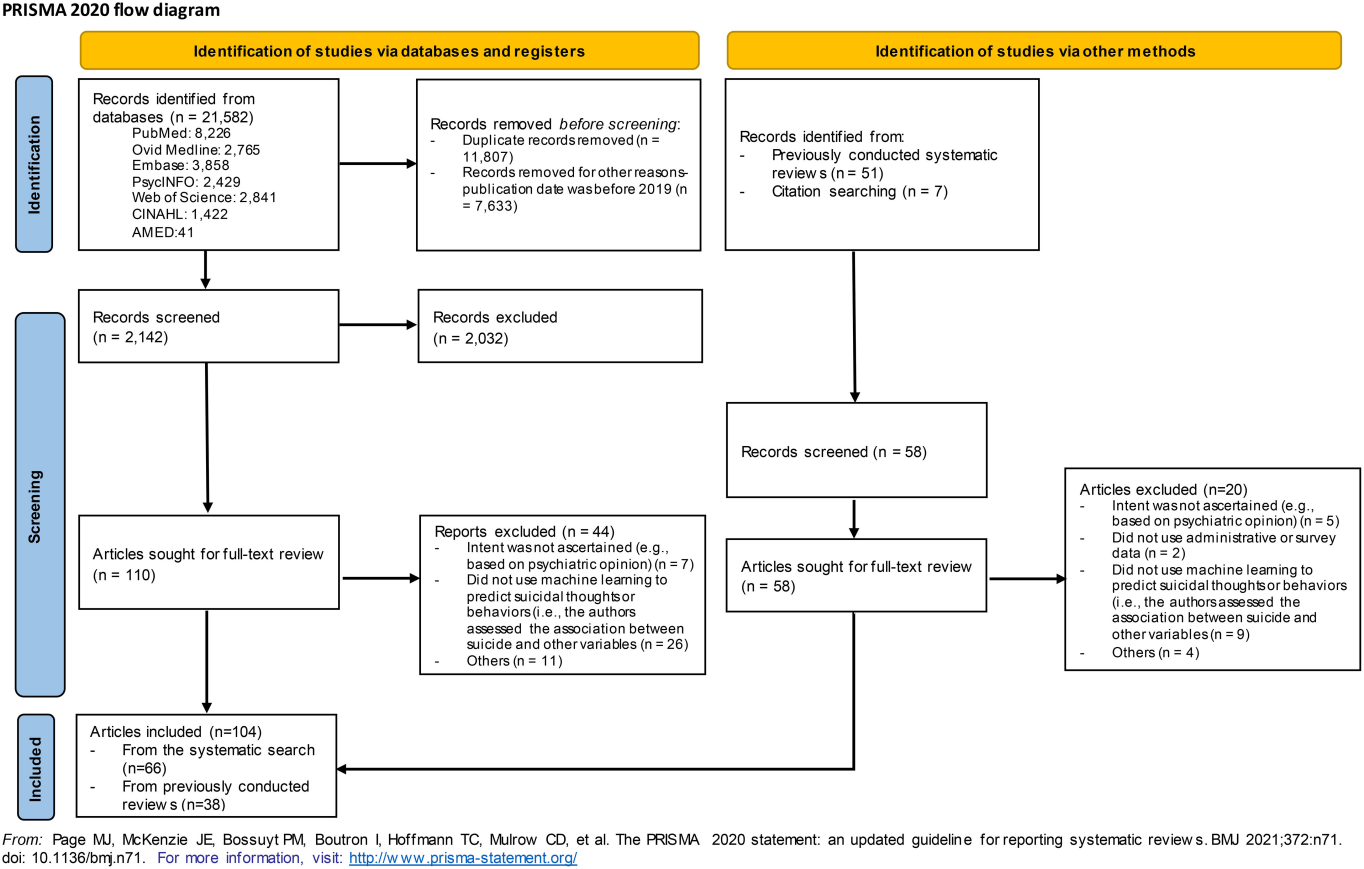
Flow chart.

The search strategy identified a total of 2,200 unique records, of which 168 full-text articles were retrieved (**Figure 1**). After full-text assessment, 64 were excluded (the reasons for their exclusion are presented in **Appendix 2**), and 104 articles were included.

### Study characteristics

Over 85% (n=90) of eligible studies were published since 2017. The majority of studies were from the United States (n=43, 41.4%), followed by South Korea (n=17, 16.4%) and Canada (n=5, 4.81%). Twenty-six studies used machine learning to predict suicidal thoughts, 55 studies aimed to predict suicide attempts, and 19 studies predicted death by suicide (it should be noted that some studies predicted more than one suicide outcome of interest). Survey data (57.69%) was used more often than administrative data (n=41, 39.4% and n=60, 57.7%, respectively), and three (2.9%) studies used both types of data. Over 60% (n=63) used a longitudinal study design, while the remainder used a cross-sectional study design (one study did not provide sufficient information to determine the study design). Existing studies applied various machine learning methods, most of which were supervised learning techniques, including logistic regression (n=38, 17.9%), random forest (n=37, 17.4%) and decision tree (n=30, 14.1%). K-fold cross-validation techniques (with K=5 or 10) were the most used model’s performance evaluation methods (n=67, 64%), followed by the hold-out method (n=12, 12%) and bootstrap-based optimism correction methods (n=6, 6%). 19 (18%) studies did not provide the algorithm validation method. Cross-validation is usually the preferred method because it allows the model to train on multiple train-test splits, better indicating the model performance on unseen data. Please see **Supplementary Table 1** for more details on the characteristics of each study included in the **Supplemental Material**.

### Performance of applied machine learning methods

Different metrics were used to evaluate model performance; of 104 included studies, a total of 84 studies reported the AUC (Mean = 0.807, 95% CI: 0.787-0.826, SD = 0.088, Median = 0.818, Range = 0.588-0.987). Additionally, 49 studies reported Accuracy (Mean = 0.822, 95% CI: 0.782-0.863, SD = 0.168, Median = 0.838, Range = 0.250-0.996). Sixty-four studies reported sensitivity or prediction of the positive class (Mean = 0.682, 95% CI: 0.628-0.735, SD = 0.215, Median = 0.742, Range = 0.128-1), and 59 studies described specificity or prediction of the negative class (Mean = 0.809, 95% CI: 0.765-0.853, SD = 0.168, Median = 0.820, Range = 0.25-1), separately. Positive predictive value (PPV), or the percentage of positively categorized cases that were actually positive was assessed in 46 articles (Mean = 0.412, 95% CI: 0.315-0.508, SD = 0.325, Median = 0.385, Range = 0.02-1), and 36 studies reported negative predictive value (NPV), the proportion of negatively categorized cases that were actually negative (Mean = 0.911, 95% CI: 0.875-0.947, SD = 0.107, Median = 0.963, Range = 0.600-0.999).

**Figure 2:**
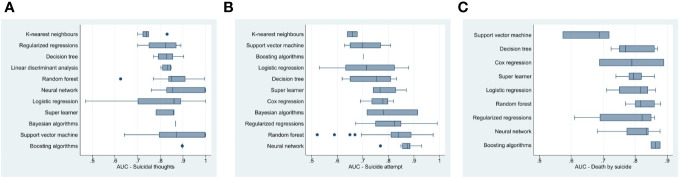
Box plot of machine learning method area under the receiver operating characteristic curve for **(A)** suicidal thoughts, **(B)** suicide attempt and **(C)** death by suicide.

**Figure 3:**
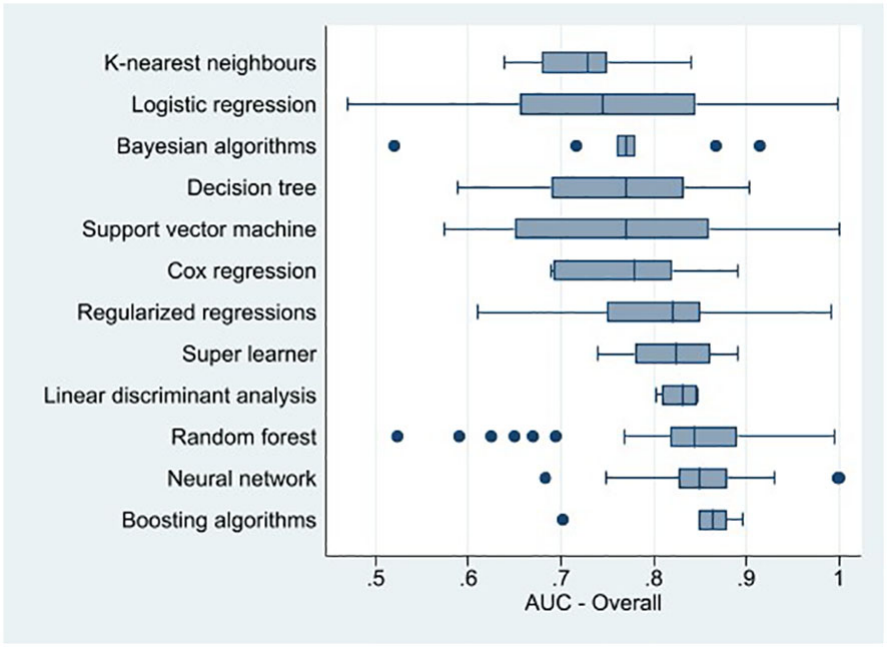
Box plot of machine learning method area under the receiver operating characteristic curve for suicidal thoughts and behaviors combined.

These results suggest that, on average, machine learning algorithms achieved good prediction of suicidal thoughts and behaviors. However, machine learning methods’ predictive power appears to differ across suicide outcomes. **Figures 2**, **3** present the distribution of the AUCs by machine learning methods for suicidal thoughts, suicide attempts, and death by suicide, as well as all suicide outcomes combined in a box plot. Although the figure displays a few outliers, the AUCs for boosting algorithms were closer to 0.9 than the other algorithm’s AUCs when predicting suicidal thoughts, death by suicide, and all suicide outcomes combined (**Figure 3**). Neural network algorithm AUCs were concentrated around the highest AUC value in predicting suicide attempts (with nearly all the AUC values between 0.8 and 0.9, indicating good prediction). This algorithm was the second best at predicting death by suicide and all suicide outcomes combined. Random forest and support vector machine were the second-best algorithms at predicting suicide attempts and suicidal thoughts, respectively, with the majority of the AUCs between 0.8 and 0.9. It should be noted that the AUCs of the support vector machine were around 0.7 (the smallest) for death by suicide and suicide attempt prediction. The AUCs of the K-nearest neighbors algorithm were between 0.6 and 0.79 for suicidal thoughts, suicide attempts, and all suicide outcomes combined.

In addition, logistic regression displayed relatively lower AUCs and more variability than the other machine learning methods overall, except for death by suicide. However, boosting algorithms and neural network performed better than the logistic regression at predicting death by suicide (**Figure 2**). The mean AUCs in **Table 1** indicate that overall, logistic regression, neural network, boosting algorithms, neural network, K-nearest neighbors, regularized regressions, and random forest had an average AUC of between 0.80 and 0.89 (i.e., good prediction of suicidal thoughts and behaviors). **Table 1** also shows heterogeneity in the performance of machine learning methods across suicide outcomes.

**Table 1:** The mean AUC of all the models (95% confidence interval) for predicting suicide outcomes by study design, data source, sample size, and type of machine learning methods.

	Suicidal thoughts	Suicide attempt	Death by suicide	Overall
Study design				
Cross-sectional	0.844 (0.821 - 0.868)	0.808 (0.777 - 0.840)	0.750 (0.671 - 0.828)	0.801 (0.780 - 0.823)
Longitudinal	0.833 (0.798 - 0.867)	0.790 (0.774 - 0.807)	0.799 (0.780 - 0.818)	0.800 (0.788 - 0.812)
Data source				
Administrative	0.915 (0.880 - 0.949)	0.792 (0.774 - 0.810)	0.799 (0.780 - 0.818)	0.816 (0.802 - 0.830)
Survey	0.801 (0.777 - 0.826)	0.802 (0.777 - 0.826)	0.750 (0.671 - 0.828)	0.788 (0.771 - 0.805)
Administrative & Survey	–	0.788 (0.746 - 0.829)	–	0.788 (0.746 - 0.829)
Total Sample size				
≤1,000	0.847 (0.815 - 0.879)	0.764 (0.735 - 0.793)	–	0.768 (0.744 - 0.792)
1,001-10,000	0.839 (0.810 - 0.867)	0.787 (0.770 - 0.804)	0.777 (0.709 - 0.846)	0.805 (0.789 - 0.821)
>10,000	0.771 (0.640 - 0.903)	0.859 (0.826 - 0.892)	0.794 (0.774 - 0.814)	0.816 (0.798 - 0.833)
Target sample size				
≤200	0.885 (0.845 - 0.925)	0.775 (0.756 - 0.794)	0.797 (0.732 - 0.862)	0.797 (0.780 - 0.815)
201-1,000	0.803 (0.780 - 0.826)	0.829 (0.792 - 0.866)	0.767 (0.730 - 0.804)	0.794 (0.776 - 0.813)
>1,000	0.771 (0.640 - 0.901)	0.828 (0.796 - 0.859)	0.813 (0.792 - 0.834)	0.813 (0.795 - 0.831)
Machine learning method				
Bayesian algorithms	–	0.804 (0.552 - 1.055)	–	0.764 (0.698 - 0.829)
Boosting algorithms	0.767 (0.682 - 0.853)	–	0.864 (0.678 - 1.050)	0.775 (0.694 - 0.855)
Cox regression	0.828 (0.794 - 0.861)	0.741 (0.685 - 0.797)	0.789 (-0.491 - 2.069)	0.762 (0.731 - 0.793)
Decision tree	0.746 (0.699 - 0.793)	0.660 (0.609 - 0.711)	0.795 (0.713 - 0.877)	0.729 (0.682 - 0.777)
K-nearest neighbors	0.828 (0.793 - 0.864)	–	–	0.828 (0.793 - 0.864)
Linear discriminant analysis	0.794 (0.686 - 0.902)	0.717 (0.673 - 0.761)	–	0.746 (0.713 - 0.778)
Logistic regression	0.883 (0.780 - 0.985)	0.866 (0.828 - 0.905)	0.800 (0.764 - 0.835)	0.850 (0.819 - 0.881)
Neural network	0.860 (0.822 - 0.899)	0.842 (0.814 - 0.870)	0.808 (0.755 - 0.861)	0.846 (0.826 - 0.866)
Random forest	0.810 (0.679 - 0.941)	0.816 (0.793 - 0.839)	0.828 (0.793 - 0.863)	0.804 (0.784 - 0.825)
Regularized regressions	0.833 (0.719 - 0.948)	0.790 (0.718 - 0.862)	0.776 (0.728 - 0.825)	0.819 (0.793 - 0.846)
Super learner	0.862 (0.720 - 1.005)	0.712 (0.616 - 0.808)	0.799 (0.756 - 0.841)	0.771 (0.699 - 0.843)
Support vector machine	0.862 (0.720 - 1.005)	0.712 (0.616 - 0.808)	0.660 (0.471 - 0.849)	0.771 (0.699 - 0.843)

AUC, Area under the receiver operating characteristic curve.

The symbol "-" means no data is available to compute the summary statistics in the cell.

### The average performance of machine learning methods for predicting suicidal thoughts and behaviors by study design, data source, and sample size

The performance of machine learning methods at predicting suicidal thoughts and behaviors, on average, was similar across the type of study design and suicide outcomes, with mean AUCs ranging from 0.75 to 0.84 (**Table 1**). However, machine learning methods achieved the highest average AUC (0.915, 95% CI: 0.880-0.949) at predicting suicidal thoughts with administrative data (vs 0.801, 95% CI: 0.777-0.826 with survey data). The average performance of machine learning methods was similar in predicting death by suicide and suicide attempt across the two data sources, with mean AUCs between 0.75 and 0.80. Overall, the prediction of suicidal thoughts and suicide behaviors displayed higher AUCs on average as the sample size increased (**Table 1**). Similar results were found with the target sample size (i.e., the sample of individuals with suicidal thoughts or suicide behaviors).

### The best-performing machine learning methods average performance in predicting suicidal thoughts and behaviors by study design, data source, and sample size

Overall, the average best-performing methods for higher AUC were support vector machine, random forest, boosting algorithms, neural network, regularized regressions and super learner with AUCs greater than 0.8 (**Table 2**). Logistic regression had the lowest AUC (0.789, 95% CI: 0.737-0.841). However, logistic regression performed better than the support vector machine in the prediction of suicide attempts. Regarding suicidal thoughts, the best-performing method remained the support vector machine (AUC=0.930, 95% CI: 0.040-1.819) on average. **Table 2** also indicated that the performance of the best-performing models was, on average, similar across the study design, the data source, and the sample size for all the suicide outcomes. However, we found no evidence of an upward trend of AUCs over time, suggesting that our study did not observe the positive influence of technological and model improvements over time on the machine learning model’s predictive power.

**Table 2:** The mean AUC of the best-performing models (95% confidence interval) for predicting suicide outcomes by study design, data source, sample size, and type of machine learning methods.

	Suicidal thoughts	Suicide attempt	Death by suicide	Overall	Best-performing algorithms
Study design					
Cross-sectional	0.884 (0.859 - 0.909)	0.866 (0.822 - 0.911)	0.815 (0.014 - 1.615)	0.862 (0.835 - 0.889)	Regularized regressions
Longitudinal	0.846 (0.799 - 0.892)	0.836 (0.813 - 0.858)	0.827 (0.798 - 0.854)	0.829 (0.813 - 0.846)	Support Vector Machine
Data source					
Administrative	0.885 (0.801 - 0.968)	0.829 (0.783 - 0.875)	0.826 (0.780 - 0.854)	0.838 (0.816 - 0.861)	Support vector machine
Survey	0.849 (0.819 - 0.878)	0.857 (0.830 - 0.884)	0.815 (0.014 - 1.615)	0.842 (0.822 - 0.862)	Regularized regressions
Administrative & Survey	–	0.822 (0.695 - 0.950)	–	0.822 (0.695 - 0.950)	Regularized regressions
Total Sample size					
≤1,000	0.882 (0.818 - 0.947)	0.847 (0.799 - 0.894)	–	0.840 (0.804 - 0.792)	Regularized regressions
1,001-10,000	0.874 (0.845 - 0.904)	0.826 (0.801 - 0.851)	0.824 (0.669 - 0.979)	0.841 (0.819 - 0.863)	Support vector machine
>10,000	0.771 (0.640 - 0.903)	0.874 (0.828 - 0.921)	0.825 (0.795 - 0.856)	0.839 (0.815 - 0.862)	Regularized regressions
Target sample size					
≤200	0.910 (0.873 - 0.947)	0.838 (0.810 - 0.866)	0.819 (0.645 - 0.993)	0.843 (0.819 - 0.867)	Support vector machine
201-1,000	0.845 (0.822 - 0.868)	0.859 (0.811 - 0.906)	0.831 (0.787 - 0.874)	0.844 (0.821 - 0.866)	Regularized regressions
>1,000	0.771 (0.640 - 0.901)	0.862 (0.793 - 0.930)	0.822 (0.771 - 0.873)	0.829 (0.800 - 0.859)	Gradient boosting
Machine learning method					
Bayesian algorithms	–	–	–	0.764 (0.698 - 0.829)	–
Boosting algorithms	–	–	0.864 (0.678 - 1.050)	0.864 (0.827 - 0.900)	–
Cox regression	–	–	0.789 (-0.491 - 2.069)	0.762 (0.731 - 0.793)	–
Decision tree	–	0.760 (0.252 - 1.268)	–	0.729 (0.682 - 0.777)	–
K-nearest neighbors	–	–	–	–	–
Linear discriminant analysis	–	–	–	–	–
Logistic regression	0.812 (0.569 - 1.054)	0.823 (0.701 - 0.945)	0.788 (0.630 - 0.945)	0.789 (0.737 - 0.841)	–
Neural network	0.823 (0.676 - 0.970)	0.858 (0.750 - 0.965)	0.838 (0.741 - 0.935)	0.841 (0.803 - 0.879)	–
Random forest	0.874 (0.846 - 0.901)	0.879 (0.848 - 0.909)	0.841 (0.801 - 0.881)	0.870 (0.852 - 0.889)	–
Regularized regressions	0.795 (-0.412 - 2.002)	0.851 (0.807 - 0.894)	0.805 (0.100 - 1.511)	0.841 (0.801 - 0.879)	–
Super learner	0.860 (0.720 - 1.005)	0.802 (0.708 - 0.896)	–	0.835 (0.796 - 0.875)	–
Support vector machine	0.930 (0.040 - 1.819)	0.712 (0.616 - 0.808)	–	0.877 (0.589 - 1.164)	–

AUC, Area under the receiver operating characteristic curve.

The symbol "-" means no data is available to compute the summary statistics in the cell.

### The most important predictive risk factors of suicidal thoughts and behaviors reported in the included studies

The best-performing algorithm was used in each study to identify important predictive risk factors for suicide outcomes. The methods used depend on the algorithms. For support vector machine, decision tree, boosting algorithms and random forest to evaluate the importance of each predictor, the criteria used was the mean decrease in accuracy values, which represents the reduction in accuracy if a predictor were randomly permuted (also known as permutation feature importance methods). The most important predictors had a more considerable mean decrease in accuracy. For regularized regressions, the magnitude of the estimated weights associated with a predictor indicated how influential the predictor is in predicting the outcome. The neural networks also used weight-based methods – predictors with higher weights contributed more to the final predictions of the model.

#### Administrative data and survey studies

*Administrative.* Among studies that aimed to predict suicidal thoughts using administrative data, Peis et al. (17) and McKernan et al. (18) showed that mental illness, related inpatient utilization, and previous suicidal thoughts and attempt(s) are common risk factors in addition to some social factors like shared residence and living with offspring or siblings. Some somatic factors like low levels of free thyroxine, free triiodothyronine, temporary disability, feeling heart race/pound, and polysomatic symptoms (fatigue, dizziness, weakness) were also reported as risk factors for suicidal thoughts (17–20). Studies that used administrative data to predict suicide attempts identified the most important risk factors to be age (21–24), history of suicidal thoughts and behaviors (23–26), mental health conditions like anxiety, depression, helplessness, hopelessness and substance use (18, 21, 24–28), and having an emergency room visit or inpatient admission (18, 22, 26, 28). When the aim was to predict death by suicide using administrative data, the most important risk factors were record/indication of mental or behavioral disorders such as schizophrenia, antipsychotic medication use, depression, anxiety, stress disorders, and alcohol use (26, 29–33), followed by a prior suicide attempt or self-harm (26, 29, 30, 34), and age (30–33, 35). Four studies predicted suicidal thoughts and behaviors combined, and based on their findings, schizophrenia, personality disorders (borderline), depressive disorder, substance use disorder, family history of these disorders, related medications (such as antipsychotics and antidepressants), and intentional self-harm were the most important risk factors (36, 37) (see **Supplementary Table 2**).

*Survey.* In studies utilizing survey data, depression (sadness, hopelessness) (38–44), anger attacks (44), anxiety (41), perceived burdensomeness (38, 45), post-traumatic stress disorder (43), self-esteem (39, 42), perceived stress level (41, 46), history of suicidal ideation, attempt or familial history of suicide (38, 40, 44), and hours of sleep (40, 42) were the most important predictors identified for suicidal thoughts. In another study, Burke et al. (47) found non-suicidal self-injury (NSSI), depression, desire to cease NSSI, NSSI likelihood, and number of NSSI scars, and revenge function of NSSI as factors predictive of suicide plans when using survey data. Kuroki et al. (48) found anxiety disorder, depressive disorder, family cohesion, and family conflict to be important risk factors for suicidal ideation. Some studies predicted suicidal thoughts and suicide attempts together. However, the identified risk factors were similar to those mentioned (49–51) (**Supplementary Table 2**). Studies predicting suicide attempts found that current or past suicide plans (47, 52–55), suicidal ideation (54–58), suicide attempt(s) (59), non-suicidal self-injury (47, 55), and positive familial or friend history of suicide (57, 58, 60–62) were the established risk factors. In addition, history of mental or personality disorders such as depression (sadness/hopelessness) (45, 54, 60, 63–67), anxiety (68), was bullied or violated (57, 66, 67), borderline personality disorder (56, 69, 70), drug abuse or dependence (62, 63, 67, 71), affective dyscontrol (72), impulsivity (56, 60), post-traumatic stress disorder (69, 73), number of hospitalizations (70, 71, 73), demographic characteristics (such as age (64–66), and being female (57, 63, 68)), alcohol drinking (54, 57, 61, 64, 66), smoking (57, 63, 64, 66) were the other important risk factors for predicting suicide attempt. Regarding death by suicide, Choi et al. (74) found anxiety, depression, resilience, and self-esteem as predictive factors when using survey data.

#### Study populations

*Adolescents*. Eleven studies assessed predictive risk factors of suicidal thoughts and behaviors in children or adolescents (38, 40, 49, 50, 58, 63, 66, 67, 75, 76). Czyz et al. (38) and Hill et al. (40) reported depressive symptoms and a history of suicidal ideation or suicide attempts as risk factors for suicidal thoughts among adolescents. Further, the existing studies identified sadness or hopelessness (63, 66, 67), stress level (66, 75), number of lifetime mental disorders (59), violence or fighting (63, 66, 67), substance abuse (63, 66), cigarette smoking (63, 66), alcohol drinking (66), prior suicide attempt(s) (59), suicidal ideation (58), familial life (58, 66, 75), and demographic factors such as sex (63, 66), age (66), and belonging to a minority group (58, 63) as risk factors for suicide attempt. Three studies (49, 50, 76) investigated predictive risk factors of suicidal thoughts and suicide attempts together, and their findings were similar to the aforementioned factors (**Supplementary Table 2**).

*Soldiers*. Seven studies aimed to predict suicidal thoughts and behaviors among soldiers (19, 43, 44, 53, 68, 77, 78), all of which were conducted in Canada and the United States. Depression (43), post-traumatic stress disorder (43), sexual harassment in females (43), mental disorders (43, 44), taking any medication for mental disorders (19), somatic complaints (43) [including upset stomach during last attack (19) or feeling heart race/pound (19)], past suicidal ideation (44), and violence during deployment (44) were found to be important risk factors for suicidal thoughts (**Supplementary Table 2**). History and number of mental disorders (68, 77), anxiety disorders (68), self-reported lifetime suicide plan (68), military service factors (77, 78), and demographic characteristics (i.e., sex (68), age (77, 78), and racial/ethnic minority status (77, 78)) as important risk factors for suicide attempt.

*Elderly*. In those studies conducted among an elderly population, quality of life (39, 46), restriction of activity (46), income level (46), stress level (46), depression (39), self-esteem (39), satisfaction with family relationships (39), and health status (39) were found to be risk factors for suicidal thoughts (**Supplementary Table 2**). Further, Cho et al. (33) found that history of taking benzodiazepines, body mass index, age, and history of taking sleeping pills were significant risk factors for death by suicide in an elderly population.

*Persons with mental health disorders*. Researchers found that in patients with obsessive-compulsive disorder, previous suicide plans or thoughts, lifetime depressive episodes, and intermittent explosive disorder were risk factors for suicide attempts (25). Among individuals with substance use disorders, males with a brief psychotic disorder diagnosis or antipsychotic prescription or females older than 30 with a ‘poisoning diagnosis’ had a higher risk of death by suicide (31). Antipsychotic and antidepressant medications, a diagnosis of autistic disorder, schizophrenic disorder, and substance use disorder were reported by Fan et al. (37) as risk factors for suicidal thoughts, suicide attempts, or death by suicide among patients with post-traumatic stress disorder and bipolar disorder. Hettige et al. (71) reported the number of hospitalizations, duration of illness, childhood trauma (such as physical and emotional abuse), and substance abuse or dependence as risk factors for suicide attempts among individuals with schizophrenia. In patients with lifetime major depressive episodes, previous suicide attempts (69), borderline personality disorder (69), and hospitalization due to depressive symptoms (69) were identified as risk factors for suicide attempts. Low free triiodothyronine, low free thyroxine, severity of depressive symptoms, and work status were found to be important risk factors for suicidal thoughts (20). Age (21, 23, 79), history of mental disorder (22) (such as anxiety (21)), having a suicide plan, intent or positive familial suicidal history (23, 52, 61), number of outpatient, inpatient, and emergency room visits (22), and educational level (21, 79) were identified as predictive risk factors for suicidal thoughts or suicide attempt among individuals with a history of suicide attempt. Based on the studies that aimed to predict suicidal thoughts and behaviors among individuals with a mood disorder, depression (hopelessness and helplessness) (27, 56, 72, 73, 80), borderline personality disorder (56, 80), prior suicidal ideation, attempt or history of suicide in their family (26, 56, 80), substance abuse or dependence (26, 62, 73), aggression (56), affective dyscontrol (72), loss of cognitive control (72), history of psychosis (73), post-traumatic stress disorder comorbidity (73), having an emergency department visit or inpatient hospitalization with a high-lethality diagnosis (28), and a history of physical illnesses (81) were identified as risk factors for suicide attempts. In addition, hospitalized for schizophrenia-spectrum and bipolar disorders (34), previous self-harm (34, 80) or suicidal attempt or thoughts (26), prior hospitalization or emergency mental health care (26, 80) and substance abuse (26) were mentioned as risk factors for death by suicide in this group (**Supplementary Table 2**).

*General population.* Fourteen studies sought to predict the most important risk factors of suicidal thoughts and behaviors among the general population. Kuroki et al. (48), Ryu et al. (41), and Peis et al. (17) found that depression (41, 48), anxiety (41, 48), stress (41), previous suicidal thoughts or suicide attempts (17), and shared residence or living with siblings or offspring (17) were risk factors for suicidal thoughts (**Supplementary Table 2**). Depression (45, 60, 64, 65), impulsivity (60), borderline personality disorder (69), post-traumatic stress disorder (69), alcohol use disorders identification test (AUDIT) score (64) or frequency of drinking (64), previous history of suicide attempt (82) or suicide among family members (60) or friends (82), lower family support or higher familial conflict (48), substance use in the previous two weeks (82), and demographic characteristics including age (64, 65, 83), lower educational level (65, 82, 83), and being female (82, 83) were important when predicting suicide attempt.

Also, age (32, 35), sex (30, 35), depression (29, 30, 74), anxiety (29, 74), self-esteem (74), alcohol consumption (32), prior self-harm (29) or suicide attempt (30), stress disorders (30), and schizophrenia in females or antipsychotics in males were reported as risk factors for death by suicide among the general population.

### Risk of bias

Based on PROBAST, two articles had a high risk of bias, twenty-seven had an unclear risk of bias (mostly due to unclear information in the predictors, outcome, and analysis domains), and the others were classified as having a low risk of bias. Regarding applicability, all included articles were categorized as being of low concern. The results of these evaluations are summarized in **Appendix 3**.

## Discussion

The current systematic review summarized studies that applied machine learning methods to administrative and/or survey data to predict suicidal thoughts and behaviors. This review included 104 articles, all published within the last 25 years. Although the predictive power of models differed across suicide outcomes (i.e., suicidal thoughts, suicide attempt, and death by suicide), overall, machine learning algorithms achieved good prediction of suicidal thoughts and behaviors when using administrative and/or survey data. Many machine learning algorithms performed better than logistic regression in predicting suicide outcomes, including boosting algorithms and neural networks. Additionally, studies with greater total and target sample sizes reported higher prediction accuracies. We also found that the risk factors for suicidal thoughts and behaviors appear to differ depending on the data source and the population under study.

Considering that many individuals who have attempted suicide or died by suicide visited their physician or emergency room prior to (22, 61, 84), the application of machine learning techniques using administrative data is a promising tool, as it could help improve early detection of individuals who are at high risk for suicidal behaviors. There is also the potential for such techniques to relieve an already over burden healthcare system by providing clinicians with a tool for suicide risk identification. In fact, it has been shown that risk stratification via electronic medical records data predicts suicide risk better than clinical evaluation (28). Thus, a combination of machine learning and routine triage could optimize clinical decision-making and conserve human and financial resources. Further, the application of machine learning techniques on survey data from a variety of populations could be helpful in detecting and preventing potential high-risk subpopulations.

To the best of our knowledge, this is the largest (104 included studies) systematic literature review on the application of machine learning techniques to predict suicidal thoughts and behaviors. Further, this is the first review study to summarize the predictive power of machine learning in predicting suicidal thoughts and behaviors by type of method, study design, data source, and sample size and to summarize extensively the most important risk factors identified. It should be noted that even though most studies had a large sample distribution of participants, around 30% of studies had a sample size of less than 1000, which could escalate the risk of overfitting and affect the interpretation of their results (investigating a large number of risk factors in a small sample of participants). Further, the majority of studies were from the United States (>40%); thus, studies need to be conducted elsewhere. For instance, very few studies (n=5) (19, 71, 85–87) used data from Canada to predict suicidal thoughts and behaviors using machine learning. Only three of these studies used population-level data from the province of Alberta. To fill this gap and advance the application of machine learning in detecting suicide risk, more studies are needed, focusing on larger and more comprehensive population-based datasets that can facilitate complex modeling incorporating relevant risk factors.

Furthermore, very few studies (n=5) (30, 31, 36, 43, 88) stratified their analyses by sex. This represents a significant gap in the literature, as the gender paradox in suicide (i.e., women tend to attempt suicide more often, while men tend to die by suicide more often) is well-known. The gender paradox in suicide is a culture-bound phenomenon, meaning that cultural expectations about gender and suicide strongly determine both its existence and magnitude (89, 90). As such, the risk factors predictive of suicidal thoughts and behaviors will likely differ for men and women. Gender-specific analyses have the potential to identify the risk factors that may be predictive of suicidal thoughts and behaviors in one gender but not the other and thus can further inform targeted suicide prevention strategies.

Overall, the findings of the current review should prove helpful in preparing future machine learning models using survey/administrative data to predict suicide outcomes, their application in clinical decision-making, and planning prevention interventions. As indicated by the sheer number of studies available, the application of machine learning for predicting suicidal thoughts and behaviors represents an area of research which has seen significant growth. This growth has significant implications for the prevention of suicide and, thus, the reduction of the suicide mortality rate globally.

However, in many studies, the complete list of predictors used in the machine learning algorithms was not available. To advance this field, we suggest that future studies using machine learning to predict suicide outcomes enumerate all the predictors in their models (in the supplement) and the list of the most important predictors identified by the best-performing model. This will help other researchers to carefully select their predictors and investigate other variables that might improve models’ prediction. It will also help decision-makers or program planners to translate findings into more practical tools to enhance suicide prevention. Additionally, to improve the model’s performance, future studies must account for the data imbalance issues caused by the rarity of suicide outcomes using more sophisticated sampling methods such as the Synthetic Minority Over-sampling Technique (91) or ensemble learning techniques (92). Our study has revealed that most included studies using longitudinal data have failed to account for the correlation of individual observations over time. Thus, the assumption that training data is independent and identically distributed may be violated, making supervised machine learning algorithms inefficient. We recommend that future studies aiming to predict suicide outcomes with longitudinal data consider using mixed-effect machine learning algorithms. These algorithms are robust to correlated data and predict change of a longitudinal outcome with high accuracy (93). Finally, future studies need to rely on novel causal inference methods based on machine learning to help understand causal relationships between important predictors and suicide outcomes (94, 95).

## Data availability statement

The original contributions presented in the study are included in the article/**Supplementary Material**. Further inquiries can be directed to the corresponding author.

## Author contributions

NHS: Conceptualization, Investigation, Methodology, Project administration, Supervision, Validation, Visualization, Data curation, Formal analysis, Writing – original draft, Writing – review & editing. PN: Data curation, Formal analysis, Investigation, Validation, Writing – original draft. SL: Conceptualization, Investigation, Methodology, Project administration, Resources, Supervision, Validation, Visualization, Writing – original draft, Writing – review & editing.
