# Supplementary material for: The use of machine learning on administrative and survey data to predict suicidal thoughts and behaviors: a systematic review
Source: Front Psychiatry. 2024 Mar 4;15:1291362. doi: 10.3389/fpsyt.2024.1291362 (PMC10944962; doi:10.3389/fpsyt.2024.1291362)
Supplement: Supplementary file 1 [file Table_1.docx]

**Supplemental Material**

Table 1. Study characteristics, model type and predictive performance for the included studies

| **Reference** | **Study population (Country)** | **Sample Size (Overall N; Target n; Control n)** | **Study Design Data Source (Study period)** | **Suicide outcomes** | **Model performance evaluation methods** | **Model type and performance** |
| --- | --- | --- | --- | --- | --- | --- |
| Adams et al. 2021 (17) | Men with substance use disorders diagnosis (Denmark)  Women with substance use disorders diagnosis (Denmark) | (10,208; 1,985; 8,223);  (5,745; 789; 4,956) | Longitudinal Administrative (1995-2015) | Death by suicide | Cross-validation | Decision tree: Men–AUC=0.75; Random forest: AUC=0.77; Women–AUC=0.86; Random forest: AUC=0.86 |
| Agne et al. 2020 (18) | Patients with obsessive-compulsive disorder (Brazil) | (959; 104; 855) | Cross-sectional Administrative (2003-2009) | Suicide attempt | Cross-validation | Elastic net: Accuracy‎=0.86, SE‎=0.85, SP=0.87, PPV=0.45, NPV=0.98, AUC=0.95 |
| Bae et al. 2015 (19) | Middle and high school students (South Korea) | (1,910; 186; 1,744) | Cross-sectional Survey (2011) | Suicide attempt |  | Decision tree: AC‎=0.9 |
| Baca-Garcia et al. 2007 (20) | Suicide attempters 18 years or older recruited in an emergency room (Spain) | (539; 51; 488) | Cross-sectional Survey (1996-1998) | Suicide attempt (family history of suicide attempt) | Cross-validation | Logistic regression (Forward selection): AC‎=0.97, SE ‎=0.78, SP =0.99 |
| Ballester et al. 2021 (21) | 18- to 24-year-old participants living in the urban area (Brazil) | (1,069; 91; 978) | Longitudinal Survey (2007-2009 and 2012-2014) | Suicide attempt or suicidal thoughts | Cross-validation | Gradient boosting: AC‎=0.64, SE ‎=0.4, SP =0.87, PPV =0.22, NPV=0.94, AUC=0.71; Elastic net: AC‎=0.65, SE ‎=0.51, SP =0.78, PPV =0.18, NPV=0.95, AUC=0.7 |
| Barros et al. 2017 (22) | Mental health patients (Chile) | (707; 349; 358) | Longitudinal Survey (2010-2014) | Suicide attempt or suicidal thoughts | Cross-validation | Decision tree: AC‎=0.72, SE ‎=0.71, SP =0.74;  K-nearest neighbours: AC‎=0.73, SE ‎=0.74, SP =0.73;  Random forest: AC‎=0.78, SE ‎=0.78, SP =0.77;  AdaBoost: AC‎=0.76, SE ‎=0.75, SP =0.76;  Support vector machine: AC‎=0.78, SE ‎=0.77, SP =0.79 |
| Bernecker et al. 2019 (23) | U.S. Army soldiers (U.S.) | (3251; N/A; N/A);  (1,141; N/A; N/A) | Longitudinal Administrative and Survey (2011-2014) | Suicide attempt | Cross-validation | Cox regression: SE ‎=1, AUC=0.82;  Logistic regression: AUC=0.62;  Super learner: AUC=0.83 |
| Buchman-Schmitt et al. 2020 (24) | U.S. military service members (U.S.) | (1,044; 112; 932);  (758; 63; 695) | Longitudinal Survey (3-month follow-up) | Suicide attempt or suicidal thoughts |  | Logistic regression: AUC=0.65;  Logistic regression: AUC=0.73 |
| Burke et al. 2018 (25) | Undergraduate students (U.S.) | (359; 51; 308) | Cross-sectional Survey (N/A) | Suicidal thoughts (suicide ideation and suicide plan);  Suicide attempt | Cross-validation | Suicidal ideation: Elastic net: AUC=0.85; Decision tree: AUC=0.77;  Suicide plan: Elastic net: AUC=0.89; Decision tree: AUC=0.75 Suicide attempt: Elastic net: AUC=0.75; Decision tree: AUC=0.74 |
| Chen et al. 2020 (26) | Patients 18 to 39 years old visited psychiatric specialty care (Sweden) | (541,300; 1,8682; 522,618);  (541,300; 9,099; 532,201);  (541,300; 18,682; 522,618);  (541,300; 9,099; 532,201) | Longitudinal Administrative (2011-2012) | Death by suicide or attempt | Cross-validation | Super learner: SE ‎=0.47, SP =0.97, PPV =0.35, NPV=0.98, AUC=0.88;  Super learner: SE ‎=0.53, SP =0.96, PPV =0.19, NPV=0.99, AUC=0.89;  Super learner: SE ‎=0.47, SP =0.97, PPV =0.35, NPV=0.98, AUC=0.86;  Super learner: SE ‎=0.52, SP =0.96, PPV =0.18, NPV=0.99, AUC=0.88 |
| Cho et al. 2021 (27) | Elderly population (aged >=65 years) (South Korea) | (48,047; 100; 47,947) | Longitudinal Administrative (2009 - 2015) | Death by suicide | Cross-validation | Random forest: AC‎=0.83, SE ‎=0.6, SP =0.83, PPV =0.01, NPV=1, AUC=0.818 |
| Cho et al. 2020 (28) | Health insurance subscribers and medical aid recipients who had undergone medical check-ups (South Korea) | (372,813; 749; 372,064) | Longitudinal Administrative (2009-2015) | Death by suicide | Cross-validation | Random forest: AC‎=0.75, SE ‎=0.82, SP =0.75, PPV =0.01, NPV=1, AUC=0.849;  Random forest: AC‎=0.79, SE ‎=0.66, SP =0.79, NPV=1, AUC=0.818 |
| Choi et al. 2021 (29) | Young adults (between 18 and 34 years old) (South Korea) | (31,720; 306; 31,414) | Cross-sectional Survey (2018-2019) | Death by suicide | Bootstrap optimism correction | Deep neural network: AC‎=0.83, SE ‎=0.76, SP =0.83, AUC=0.878;  Logistic regression: AC‎=0.98, SE ‎=0.5, SP =0.98, AUC=0.74;  Support vector machine: AC‎=0.99, SE ‎=0.15, SP =1, AUC=0.574;  Super learner: AC‎=0.87, SE ‎=0.69, SP =0.87, AUC=0.861 |
| Choi et al. 2018 (30) | General population (South Korea) | (819,951; 2,546; 817,405) | Longitudinal Administrative (2004-2013) | Death by suicide | Cross-validation | Cox regression model: AUC=0.6884;  Support vector machine: AUC=0.687;  Deep neural network: AUC=0.683 |
| Colic et al. 2018 (31) | Veterans still serving members of the Canadian Forces and Royal Canadian Mountain Police (Canada) | (738; 331; 407) | Cross-sectional Administrative ‎(N/A)‎ | Suicidal thoughts | Cross-validation | Random forest: AUC=0.844 |
| Kessler et al. 2020 (32) | Veteran Health Administration hospital patients hospitalized for psychiatric disorders (U.S.) | (195,349; 771; 194,578) | Longitudinal Administrative (2010–2013) | Death by suicide | Cross-validation | Super learner: AUC=0.74-0.82 |
| Czyz et al. 2021 (33) | Psychiatrically hospitalized adolescents (aged 13–17) (U.S.) | (78; 64; 14) | Longitudinal Survey (2019- 2020 ) | Suicidal thoughts | Cross-validation | Super learner: SE ‎=0.81, SP =0.82, PPV =0.74, AUC=0.86;  Super learner: SE ‎=0.64, SP =0.84, PPV =0.6, AUC=0.78 |
| Ribeiro et al. 2019 (34) | Adults recruited from online forums focused on mental health, suicide, and self-injury (Worldwide) | (1,021; 772; 249);  (1,021; 50; 971) | Longitudinal Survey ‎(N/A)‎ | Suicidal thoughts;  Suicide attempt | Bootstrap optimism correction | Random forest: AUC=0.87-0.89;  Random forest: AUC=0.82-0.84 |
| Delgado-Gomez et al. ‎2016 (35) | Participants aged 18 years or older (Spain) | (902; 356; 546) | Cross-sectional Survey (1999-2003) | Suicide attempt | Cross-validation | Decision tree: AC‎=0.81, SE ‎=0.87, SP =0.86 |
| DelPozo-Banos et al. 2018 (36) | People residing in Wales at the time of their death (UK) | (6,0684; 2,604; 58,080) | Longitudinal Administrative (2001-2015) | Death by suicide | Cross-validation | Neural network: SE ‎=0.65, SP =0.82, AUC=0.8 |
| Fan et al. 2020 (37) | Patients with PTSD and bipolar disorders (U.S.) | (3,168; 205; 2,963) | Longitudinal Administrative (2004-2019) | Death by suicide or, suicide attempt or suicidal thoughts | Cross-validation | K-Nearest Neighbors: SE ‎=0.89, PPV =0.17, NPV=0.99;  Naïve Bayes: SE ‎=0.98, PPV =0.07, NPV=0.98;  Decision Tree: SE ‎=0.71, PPV =0.59, NPV=0.98;  Support vector machine: SE ‎=0.56, PPV =0.08, NPV=0.95;  Logistic regression: SE ‎=0.55, PPV =0.09, NPV=0.95;  Random forest: AC‎=0.92, SE ‎=0.83, PPV =0.91, NPV=0.99, AUC=0.956 |
| Fazel et al. ‎2019 (38) | Individuals aged 15–65 with a diagnosis of severe mental illness (Sweden) | (58,771; 494; 58,277);  (16,387; 139; 16,248) | Longitudinal Administrative (2001-2008) | Death by suicide | Hold-out | Logistic regression: SE ‎=0.58, SP =0.76, AUC=0.75;  Logistic regression: SE ‎=0.55, SP =0.75, PPV =0.02, NPV=0.99, AUC=0.71 |
| Gradus et al. ‎2020 (39) | General population (Denmark) | Men: (140,743; 10152; 130,591);  Women: (138,543; 3,951; 134,592) | Longitudinal Administrative (2018-2019) | Death by suicide | Cross-validation | Men: Decision tree: AUC=0.77;  Women Decision tree: AUC=0.87;  Men: Random forest: AUC=0.8;  Women: Random forest: AUC=0.88 |
| Gradus et al.‎ 2017 (40) | Veterans of the Iraq and Afghanistan wars (U.S.) | (1,062; 179; 883);  (1,099; 191; 908) | Cross-sectional Survey (2009) | Suicidal thoughts |  | Random forest: AUC=0.91;  Random forest: AUC=0.92 |
| Handley et al. ‎2014 (41) | Participants aged 55-85 years were randomly selected from the Hunter Region (Australia) | (2,160; 95; 2,065) | Longitudinal Survey (2004-2007) | Suicidal thoughts | Cross-validation | Decision tree: AUC=0.814: Logistic regression: AUC=0.86 |
| Harman et al. ‎ 2021 (42) | Participant families (child and parent/caregiver dyads) (U.S.) | (11,176; 1,116; 10,060);  (10,253; 193; 10,060) | Longitudinal Survey ‎(N/A)‎ | Suicidal thoughts;  Suicide attempt or suicidal thoughts | Hold-out and Cross-validation | Logistic regression: AUC=0.7;  Elastic net: AUC=0.7;  Random forest: AUC=0.77 |
| Hettige et al. 2017 (43) | Participants 18 to 75 years old and meeting schizophrenia spectrum disorders (Canada) | (345; 131; 214) | Cross-sectional Survey ‎(N/A)‎ | Suicide attempt | Cross-validation | LASSO : AC‎=0.67, SE ‎=0.64, SP =0.68, PPV =0.67, NPV=0.66, AUC=0.71;  Random forest: AC‎=0.66, SE ‎=0.45, SP =0.8, PPV =0.68, NPV=0.6, AUC=0.67;  Support vector machine: AC‎=0.66, SE ‎=0.63, SP =0.68, PPV =0.67, NPV=0.66, AUC=0.7;  Elastic net: AC‎=0.65, SE ‎=0.65, SP =0.65, PPV =0.65, NPV=0.66, AUC=0.71 |
| Hill et al. ‎2017 (44) | Adolescents (U.S.) | (4,799; 523; 4,276) | Longitudinal  Survey (1994-1996) | Suicidal thoughts | Cross-validation | Decision tree: SE ‎=0.63, SP =0.82;  Decision tree: SE ‎=0.78, SP =0.68 |
| Horvath et al.‎ 2020 (45) | Prison population (U.S.) | (353; 59; 294) | Cross-sectional  Survey ‎(N/A)‎ | Suicide attempt |  | Gradient boosting: SE ‎=0.79, PPV =0.79, AUC=0.875;  Decision tree: SE ‎=0.64, PPV =0.64, AUC=0.833;  Neural network: SE ‎=0.64, PPV =0.64, AUC=0.882;  Random forest: SE ‎=0.71, PPV =0.71, AUC=0.822;  Logistic regression: SE ‎=0.36, PPV =0.36, AUC=0.635 |
| Huang et al. ‎2020 (46) | Participants who have either engaged in NSSI and/or suicide attempt in their lifetime (Worldwide) | (371; 52; 319) | Longitudinal  Survey ‎(N/A)‎ | Suicide attempt | Bootstrap optimism correction | Logistic regression: AUC=0.76;  Random forest: AUC=0.84 |
| Huang et al. ‎2020 (47) | 18+ years old (U.S.); Participants from a Web-based study (U.S.); Participants from the Military Suicide Research Consortium Database (U.S.); Participants from the National Comorbidity Survey (U.S.); Participants from the Psychology Clinic at Florida State University (U.S.); | (285; 154; 131) (933; 633; 300);  (1,584; 755; 829);  (885; 322; 563);  (182; 78; 104); | Cross-sectional  Survey ‎(N/A)‎ | Suicide attempt | Cross-validation | Random forest: AUC=0.89; Logistic regression: AUC=0.69 Random forest: AUC=0.89; Logistic regression: AUC=0.72 Random forest: AUC=0.87; Logistic regression: AUC=0.65 Random forest: AUC=0.89; Logistic regression: AUC=0.71 Random forest: AUC=0.9; Logistic regression: AUC=0.66 |
| Ji et al. ‎ 2022 (48) | Patients with major depressive disorder (China) | (92; 44; 48) | Cross-sectional  Survey (2015-2019) | Suicide attempt | Hold-out and Cross-validation | Support vector machine: AC‎=0.94;  AdaBoost: AC‎=0.82, SE ‎=0.88, SP =0.78;  Naïve Bayes: AC‎=0.82, SE ‎=0.75, SP =0.89 |
| Jordan & McNiel 2018 (49) | Hospitalized psychiatric patients (U.S.) | (218; 69; 149) | Longitudinal  Survey (1992-1995) | Suicide attempt | Cross-validation | Decision tree: SE ‎=0.77, SP =0.57, PPV =0.43, NPV=0.85, AUC=0.72 Stepwise Logistic regression: SE ‎=0.56, SP =0.69, PPV =0.43, NPV=0.79, AUC=0.63 |
| Jordan et al. ‎2018 (50) | Primary care patients (Germany) | (6,805; 857; 5,947) | Cross-sectional  Survey (2011-2016) | Suicidal thoughts | Hold-out | Decision tree: AUC=0.856; Support vector machine: AUC=0.86; Logistic regression: AUC=0.7 |
| Jung et al. ‎2019 (51) | Korean adolescents (South Korea) | (59,984; 7,443; 52,541) | Cross-sectional Survey ‎(N/A)‎ | Suicide attempt or suicidal thoughts | Cross-validation | Logistic regression: AC‎=0.78, SE ‎=0.78, SP =0.78, PPV =0.78, NPV=0.78, AUC=0.851;  Random forest: AC‎=0.78, SE ‎=0.78, SP =0.78, PPV =0.78, NPV=0.78, AUC=0.857;  Support vector machine: AC‎=0.79, SE ‎=0.78, SP =0.79, PPV =0.79, NPV=0.79, AUC=0.853;  Neural network: AC‎=0.78, SE ‎=0.77, SP =0.78, PPV =0.78, NPV=0.77, AUC=0.851;  XGBOOST: AC‎=0.79, SE ‎=0.79, SP =0.79, PPV =0.79, NPV=0.79, AUC=0.863 |
| Kessler et al. ‎2017 (52) | U.S. Veterans (U.S.) | (2,112,008; 6,360; 2,105,648) | Longitudinal  Administrative (2008-2011) | Death by suicide | Cross-validation | Elastic net: SE ‎=0.28;  Bayesian additive regression trees: SE ‎=0.28;  AdaBoost: SE ‎=0.27;  Support vector machine: SE ‎=0.21 |
| Kessler et al. ‎2017 (53) | Soldiers with outpatient mental health visits (U.S.) | (975,057; 68; 974,989) | Longitudinal  Administrative (2004-2009) | Death by suicide | Cross-validation | Elastic net: AUC=0.75 |
| Kessler et al. ‎2015 (54) | Soldiers with inpatient treatment of a psychiatric disorder (U.S.) | (40,820; 68; 40,752) | Longitudinal  Administrative (2004-2009) | Death by suicide | Cross-validation | Logistic regression: PPV =0.5, AUC=0.84;  Elastic net: PPV =0.53, AUC=0.85;  Cox regression: PPV =0.62, AUC=0.89 |
| Lee et al. ‎2021 (55) | Korean adolescents (South Korea) | (57,303; 1,731; 55,572) | Cross-sectional  Survey ‎(N/A)‎ | Suicide attempt |  | Logistic regression: AC‎=0.97, SE ‎=0.62, SP =0.97, PPV =0.1, NPV=1, AUC=0.88;  Decision tree: AC‎=0.97, SE ‎=0.58, SP =0.97, PPV =0.06, NPV=1, AUC=0.82 |
| Kim et al. ‎2021 (56) | Adolescent Psychiatric Patients(South Korea) | (124; 44; 80) | Cross-sectional  Administrative (2011-2019) | Suicidal thoughts | Cross-validation | Logistic regression: AC‎=0.89, SE ‎=0.77, SP =0.96, PPV =0.91, NPV=0.88, AUC=0.891;  Random forest: AC‎=0.89, SE ‎=0.92, SP =0.88, PPV =0.8, NPV=0.95, AUC=0.936;  Neural network: AC‎=0.78, SE ‎=0.77, SP =0.79, PPV =0.67, NPV=0.86, AUC=0.827;  Support vector machine: AC‎=0.89, SE ‎=0.85, SP =0.92, PPV =0.85, NPV=0.92, AUC=0.881;  XGBOOST: AC‎=0.86, SE ‎=0.92, SP =0.83, PPV =0.75, NPV=0.95, AUC=0.897 |
| Kim et al. ‎2021 (57) | College students (South Korea) | (7,824; 404; 7,420) | Cross-sectional  Survey ‎(N/A)‎ | Suicide attempt |  | Random forest: AC‎=0.95, AUC=0.851;  K-nearest neighbors: AC‎=0.95, AUC=0.639;  Random forest: AC‎=0.93, AUC=0.844;  K-nearest neighbors: AC‎=0.92, AUC=0.722 |
| Kim et al. ‎2021 (58) | College students (South Korea) | (8,761; 742; 8,019) | Longitudinal  Survey (2013-2015) | Suicidal thoughts |  | K-nearest neighbors: AC‎=0.93, SE ‎=0.28, SP =0.99, PPV =0.63, NPV=0.94, AUC=0.75;  Linear discriminant analysis: AC‎=0.94, SE ‎=0.43, SP =0.99, PPV =0.74, NPV=0.95, AUC=0.846;  Random forest: AC‎=0.94, SE ‎=0.44, SP =0.99, PPV =0.8, NPV=0.95, AUC=0.843 |
| Kuroki & Tilley 2012 (59) | Asian Americans (U.S.) | Attempt: (2,095; 56; 1,904);  Ideation: (2,095; 135; 1,960) | Cross-sectional  Survey (2002-2003) | Suicide attempt;  Suicidal thoughts | Cross-validation | Attempt: Decision tree: SE ‎=0.75, SP =0.39, PPV =0.39, NPV=0.75;  Suicidal thoughts: Random forest: SE ‎=0.72, SP =0.76, PPV =0.23, NPV=0.96 |
| Lee et al. ‎2019 (60) | Adolescents (South Korea) | (42,814; 970; 41,844) | Cross-sectional  Survey (2017) | Suicide attempt |  | Generalized linear model: AUC=0.85 |
| Walsh et al.‎ 2018 (61) | Adolescents with self-injury medical claims (U.S.) | (1,470; 974; 496);  (8,033; 974; 7,059);  (26,055; 974; 25,081) | Longitudinal  Administrative (1998-2015) | Suicide attempt | Bootstrap optimism correction | Random forest: AUC=0.85;  Random forest: AUC=0.90;  Random forest: AUC=0.97 |
| Lin et al.‎ 2020 (62) | Military men and women aged 18-50 years (Taiwan) | (3,546; 128; 3,418);  (3,546; 42; 3,504) | Longitudinal  Administrative ‎(N/A)‎ | Suicidal thoughts | Cross-validation | Target n=128:  Logistic regression: AC‎=1, SE ‎=1, SP =1, AUC=0.999 Decision tree: AC‎=0.98, SE ‎=0.78, SP =0.99, AUC=0.884 Random forest: AC‎=0.99, SE ‎=0.87, SP =0.99, AUC=0.977 Gradient boosting: AC‎=0.99, SE ‎=0.88, SP =0.99, AUC=0.982 Support vector machine: AC‎=1, SE ‎=1, SP =1, AUC=1 Multilayer Perceptron: AC‎=1, SE ‎=1, SP =1, AUC=1;  Target n=42:  Logistic regression: AC‎=1, SE ‎=1, SP =1, AUC=0.999 Decision tree: AC‎=0.99, SE ‎=0.79, SP =0.99, AUC=0.904 Random forest: AC‎=1, SE ‎=0.91, SP =1, AUC=0.995 Gradient boosting: AC‎=1, SE ‎=0.91, SP =0.99, AUC=0.987 Support vector machine: AC‎=1, SE ‎=1, SP =1, AUC=0.999 Multilayer Perceptron: AC‎=1, SE ‎=1, SP =1, AUC=0.999 |
| Lin et al. 2022 (63) | Suicide attempters with at least one psychiatric visit (Taiwan) | (523; 238; 285) | Longitudinal  Administrative ‎(N/A)‎ | Multiple suicide attempts |  | 60 predictors:  Decision tree: AC‎=0.92, SE ‎=0.91, SP =0.92 Neural network: AC‎=0.95, SE ‎=0.98, SP =0.93 Support vector machine: AC‎=0.9, SE ‎=0.92, SP =0.88 Super learner: AC‎=0.97, SE ‎=0.98, SP =0.97 Super learner: AC‎=0.95, SE ‎=0.98, SP =0.93 Super learner: AC‎=0.95, SE ‎=0.99, SP =0.91;  30 predictors: Decision tree: AC‎=0.92, SE ‎=0.94, SP =0.9 Neural network: AC‎=0.96, SE ‎=0.98, SP =0.94 Support vector machine: AC‎=0.88, SE ‎=0.87, SP =0.89 Super learner: AC‎=0.98, SE ‎=0.99, SP =0.98 Super learner: AC‎=0.96, SE ‎=0.98, SP =0.94 Super learner: AC‎=0.96, SE ‎=0.99, SP =0.94 |
| Lopez-Castroman et al. ‎2011 (64) | Suicide attempters from Emergency Department (Spain and France) | (1,349; 617; 732) | Cross-sectional  Administrative (1994 - 2006) | Suicide attempt | Cross-validation | Markov Blanket: AUC=0.7167 |
| Lyu JC & Zhang‎ 2019 (65) | Chinese rural residents (China) | (1,318; 659; 659) | Longitudinal  Survey (2012-2014) | Suicide attempt |  | Logistic regression: SE ‎=0.8, SP =0.84;  Neural network: SE ‎=0.68, SP =0.94, PPV =0.86, NPV=0.84, AUC=0.85 |
| Machado et al.‎ 2021 (66) | U.S. civilian noninstitutionalized population (U.S.) | (32,700; 200; 32,500);  (6,350; 150; 6,200) | Longitudinal  Survey (3-year follow-up) | Suicide attempt | Cross-validation | All participants N=32,700: Elastic net: AC‎=0.82, SE ‎=0.75, SP =0.89, PPV =0.05, NPV=1, AUC=0.89;  Random forest: AC‎=0.83, SE ‎=0.82, SP =0.84, PPV =0.04, NPV=1, AUC=0.89;  Neural network: AC‎=0.82, SE ‎=0.8, SP =0.84, PPV =0.03, NPV=1, AUC=0.86 Participants with lifetime major depressive episodes N=6,350: Elastic net: AC‎=0.82, SE ‎=0.77, SP =0.86, PPV =0.1, NPV=0.99, AUC=0.89;  Random forest: AC‎=0.82, SE ‎=0.84, SP =0.8, PPV =0.08, NPV=1, AUC=0.89;  Neural network: AC‎=0.81, SE ‎=0.84, SP =0.77, PPV =0.07, NPV=1, AUC=0.88 |
| Mangino et al. ‎2022 (67) | Adolescents and young adults (U.S.) | (203,663; N/A; N/A) | Cross-sectional  Survey ‎(N/A)‎ | Suicide attempt |  | Logistic regression: AC‎=0.56, SE ‎=0.52, SP =0.56, AUC=0.537;  Random forest: AC‎=0.94, SE ‎=0.92, SP =0.96, AUC=0.955;  Gradient boosting: AC‎=0.94, SE ‎=0.92, SP =0.95, AUC=0.985;  Bayesian additive regression trees: AC‎=0.92, SE ‎=0.84, SP =0.99, AUC=0.915 |
| Mann et al. ‎2008 (68) | Patients with mood, schizophrenia spectrum, or personality disorders (U.S.) | (408; 80; 210) | Longitudinal  Survey (1989-1998) | Suicide attempt | Cross-validation | Decision tree: SE ‎=0.56, SP =0.91, PPV =0.7, NPV=0.85, AUC=0.8 |
| Marcon et al. ‎2019 (69) | Medical students (Brazil) | (4,840; 432; 4,408) | Cross-sectional  Survey ‎(N/A)‎ | Suicide attempt | Cross-validation | Elastic net: SE ‎=0.75, SP =0.73, PPV =0.21, NPV=0.97, AUC=0.83 |
| McKernan et al. ‎2019 (70) | Patients with Fibromyalgia (U.S.) | (8,879; 34; 8,845);  (8,884; 96; 8,788) | Longitudinal  Administrative (1998-2017) | Suicide attempt;  Suicidal thoughts | Hold-out | LASSO: AUC=0.82;  LASSO: AUC=0.8 |
| Modai et al. ‎1998 (71) | Hospitalized psychiatric patients (Israel) | (122; 22; 100);  (41; 16; 25) | Longitudinal  Administrative ‎(N/A)‎ | Suicide attempt |  | Neural network: SE ‎=0.54, SP =0.97, PPV =0.88, NPV=0.75;  Neural network: SE ‎=0.83, SP =0.78, PPV =0.45, NPV=0.95 |
| Miché et al. 2020 (72) | Adolescents and young adults aged 14–24 years (Germany) | (2,793; 137; 2,656) | Longitudinal  Survey (1995-2005) | Suicide attempt | Cross-validation | Logistic regression: SE ‎=0.22, PPV =0.7, AUC=0.828;  LASSO: SE ‎=0.21, PPV =0.72, AUC=0.826;  Ridge regression: SE ‎=0.25, PPV =0.66, AUC=0.829;  Random forest: SE ‎=0.03, PPV =0.87, AUC=0.824 |
| Modai et al. ‎1999 (73) | Psychiatric patients (Israel) | (198; 99; 99) | Longitudinal  Survey ‎(N/A)‎ | Suicide attempt |  | Neural network: SE ‎=0.94, SP =0.9, PPV =0.92, NPV=0.96 |
| Morales et al. ‎2017 (74) | Patients with affective disorders (Chile) | (707; 349; 358) | Cross-sectional  Survey (2010-2015) | Suicide attempt or suicidal thoughts | Cross-validation | Decision tree: AC‎=0.67, SP =0.67, AUC=0.7335 |
| Na et al. ‎2022 (75) | Community-dwelling elderly aged >55 years (South Korea) | (6,410; 173; 6,237) | Longitudinal  Survey ‎(N/A)‎ | Suicidal thoughts | Cross-validation | Random forest: AC‎=0.87, SE ‎=0.75, SP =0.87, PPV =0.14, NPV=0.99, AUC=0.879 |
| Naghavi et al.‎ 2020 (76) | University students (Iran) | (573; 143; 430) | Cross-sectional  Survey (March 2020 and May 2020) | Suicide attempt or suicidal thoughts | Cross-validation | Decision tree: SE ‎=0.81, SP =0.98, PPV =0.94, AUC=0.9 |
| Nordin et al. ‎2021 (77) | Patients with depression (Malaysia) | (75; 23; 52) | Cross-sectional  Administrative (2007-2008) | Suicide attempt | Cross-validation | Logistic regression: AC‎=0.83, SE ‎=0.9, SP =0.58, PPV =0.81, NPV=0.72, AUC=0.74 Decision tree: AC‎=0.82, SE ‎=0.91, SP =0.5, PPV =0.81, NPV=0.75, AUC=0.65 Support vector machine: AC‎=0.84, SE ‎=0.92, SP =0.6, PPV =0.86, NPV=0.74, AUC=0.81 Naïve Bayes: AC‎=0.82, SE ‎=0.91, SP =0.5, PPV =0.83, NPV=0.73, AUC=0.78 K-nearest neighbours: AC‎=0.79, SE ‎=0.86, SP =0.5, PPV =0.79, NPV=0.72, AUC=0.68 Random forest: AC‎=0.87, SE ‎=0.91, SP =0.5, PPV =0.81, NPV=0.75, AUC=0.65 Bagging: AC‎=0.92, SE ‎=0.92, SP =0.53, PPV =0.89, NPV=0.76, AUC=0.87 Voting: AC‎=0.92, SE ‎=0.92, SP =0.58, PPV =0.81, NPV=0.75, AUC=0.74 |
| Oh et al. 2020 (78) | General population (South Korea) | (3788; 210; 3578) | Cross-sectional  Survey (2010-2013) | Suicidal thoughts | Cross-validation | Bayesian network: AC‎=0.76, SE ‎=0.82, SP =0.75, PPV =0.16, NPV=0.99, AUC=0.867Logistic regression: AC‎=0.79, SE ‎=0.81, SP =0.79, PPV =0.18, NPV=0.99, AUC=0.877Support vector machine: AC‎=0.81, SE ‎=0.78, SP =0.81, PPV =0.2, NPV=0.98, AUC=0.794Decision tree: AC‎=0.72, SE ‎=0.81, SP =0.71, PPV =0.14, NPV=0.99, AUC=0.843Neural network: AC‎=0.77, SE ‎=0.81, SP =0.77, PPV =0.17, NPV=0.99, AUC=0.877Logistic regression: AC‎=0.79, SE ‎=0.79, SP =0.79, PPV =0.18, NPV=0.99, AUC=0.867 |
| Oh et al. ‎2017 (79) | Patients with depression and anxiety disorders (South Korea) | (573; 39; 534);  (573; 68; 505);  (573; 163; 410) | Cross-sectional  Survey (2011- 2017) | Suicide attempt |  | Neural network: AC‎=0.94, SE ‎=0.13, SP =1, AUC=0.93 Neural network: AC‎=0.91, SE ‎=0.34, SP =0.98, AUC=0.87 Neural network: AC‎=0.87, SE ‎=0.78, SP =0.91, AUC=0.89 |
| Parghi et al. 2020 (80) | High‐risk psychiatric inpatients (U.S.) | (591; 20; 571) | Longitudinal  Survey (2016-2019) | Suicide attempt | Hold-out | Logistic regression: AC‎=0.96, AUC=0.82 Random forest: AC‎=0.98, AUC=0.878 Gradient boosting: AC‎=0.98, AUC=0.894 |
| Passos et al. ‎2016 (81) | Patients with mood disorders (U.S.) | (144; 43; 101) | Cross-sectional  Survey (2006-2010) | Suicide attempt | Cross-validation | Relevance vector machine: AC‎=0.72, SE ‎=0.72, SP =0.71, PPV =0.52, NPV=0.86, AUC=0.77 Support vector machine: AC‎=0.67, SE ‎=0.58, SP =0.71, PPV =0.46, NPV=0.8, AUC=0.65 LASSO: AC‎=0.73, SE ‎=0.56, SP =0.8, PPV =0.55, NPV=0.81, AUC=0.73 |
| Rosellini et al.‎ 2017 (82) | New U.S. Army soldiers (U.S.) | (21,832; 169; 21,663) | Cross-sectional  Survey (2011-2012) | Suicide attempt |  | Elastic net: AUC=0.74 |
| Rosellini et al. ‎2018 (83) | U.S. Army soldiers (U.S.) | (7,081; 107; 6,974) | Longitudinal  Survey (2012-2013) | Suicidal thoughts |  | Logistic regression: AUC=0.72 Super learning: PPV =0.13, AUC=0.86 |
| Seunghyong Ryu, 2019 (84) | Individuals aged over 19 years in Korea (South Korea) | (796; 397; 399) | Longitudinal  Survey (19 years) | Suicide attempt | Cross-validation | Random forest: AC‎=0.89, SE ‎=0.86, SP =0.92, PPV =0.91, NPV=0.87, AUC=0.947 |
| Ryu et al. 2018 (85) | General Population (South Korea) | (35,116; 5,814; 29,302) | Longitudinal  Survey (19 years) | Suicidal thoughts | Cross-validation | Random forest: AC‎=0.82, SE ‎=0.84, SP =0.81, PPV =0.46, NPV=0.96, AUC=0.85 |
| Sanderson et al.‎ 2020 (86) | General population (Canada) | (39,028; 3548; 35,480) | Longitudinal  Administrative (2000-2016) | Death by suicide | Cross-validation | Neural network: AC‎=0.8, SE ‎=0.72, SP =0.81, PPV =0.27, NPV=0.97, AUC=0.8419 XGBOOST: AC‎=0.82, SE ‎=0.7, SP =0.83, PPV =0.29, NPV=0.96, AUC=0.8493 |
| Sanderson et al. 2020 (87) | Patients with an ED visit for parasuicide (Canada) | (33,426; 268; 33,158) | Longitudinal  Administrative (2010-2017) | Death by suicide | Cross-validation | Logistic regression: AC‎=0.84, SE ‎=0.74, SP =0.84, PPV =0.04, NPV=1, AUC=0.8632 XGBOOST: AC‎=0.89, SE ‎=0.69, SP =0.89, PPV =0.05, NPV=1, AUC=0.8786 |
| Sanderson et al. ‎2019 (88) | General population (Canada) | (39,028; 3,548; 35,480) | Longitudinal  Administrative (2000-2016) | Death by suicide | Cross-validation | Logistic regression: AC‎=0.81, SE ‎=0.65, SP =0.83, PPV =0.27, NPV=0.96, AUC=0.8179 Neural network: AC‎=0.8, SE ‎=0.7, SP =0.81, PPV =0.27, NPV=0.96, AUC=0.8352 |
| Shaw et al.‎ 2021 (89) | American Indian/Alaska Native patients aged ≥13 years with behavioral health diagnoses (U.S.) | (47,413; 589; 46,824) | Longitudinal  Administrative (2016-2018) | Suicide attempt | Hold-out | LASSO: AC‎=0.19, AUC=0.826 |
| Shen et al.‎ 2020 (90) | Chinese medical college students (China) | (4,882; 682; 4,200) | Cross-sectional  Survey (January to March 2018) | Suicide attempt | Cross-validation | Random forest: AC‎=0.9, SE ‎=0.74, SP =0.92, AUC=0.9255 |
| Simon et al. 2018 (91) | Patients aged ≥ 13 who made specialty mental health visits and primary care visits with mental health diagnoses (U.S.) | (2,960,929; 24,133; 2,936,796);  (2,960,929; 1,240; 2,959,689) | Longitudinal  Administrative (2009-2015) | Suicide attempt;  Death by suicide | Cross-validation | Logistic regression: SE ‎=0.48, SP =0.95, PPV =0.03, NPV=1, AUC=0.853 Logistic regression: SE ‎=0.48, SP =0.95, NPV=1, AUC=0.861 |
| Simon et al. ‎2019 (92) | Patients aged ≥ 13 with outpatient visits to mental health specialty or general medical providers (U.S.) | (2,960,929; 1,240; 2,959,689);  (2,960,929; 24,133; 2,936,796) | Longitudinal  Administrative (2009-2015) | Death by suicide;  Suicide attempt |  | LASSO: AUC=0.861;  LASSO: AUC=0.853 |
| Stanley et al. 2022 (93) | U.S. Army soldiers after leaving active duty (U.S.) | (8,899; 119; 8,780) | Longitudinal  Survey (2016–2019) | Suicide attempt | Cross-validation | Super learner: AUC=0.74 |
| Tran et al. ‎2014 (94) | Patients aged ≥ 10 and had received at least one suicide risk assessment (Australia) | (7,399; 157; 7,242);  (7,399; 435; 6,964) | Longitudinal  Administrative ‎(N/A)‎ | Suicide attempt |  | LASSO: SE ‎=0.72, SP =0.72, AUC=0.79;  LASSO: SP =0.58, AUC=0.79 |
| Tubío-Fungueiriño et al. 2022 (95) | Adults with OCD (Spain) | (127; N/A; N/A) | Longitudinal  Survey ‎(N/A)‎ | Suicidal thoughts;  Suicide attempt or suicidal thoughts | Cross-validation | Linear discriminant analysis: SE ‎=0.46, SP =0.58;  Linear discriminant analysis: SE ‎=0.5, SP =0.25 |
| van Mens et al. ‎2020 (96) | Young adults (18–34 year olds) across Scotland (Scotland) | (2,428; 336; 2,092);  (2,428; 50; 2,378) | Longitudinal  Survey (one year follow up) | Suicidal thoughts;  Suicide attempt | Hold-out and Cross-validation | Suicidal thoughts: Logistic regression: SE ‎=0.49, SP =0.81, PPV =0.35, AUC=0.65 K-nearest neighbours: SE ‎=0.89, SP =0.57, PPV =0.31, AUC=0.83 Decision tree: SE ‎=0.59, SP =0.85, PPV =0.46, AUC=0.79 Random forest: SE ‎=0.6, SP =0.88, PPV =0.52, AUC=0.83 Gradient boosting: SE ‎=0.53, SP =0.9, PPV =0.53, AUC=0.82 Support vector machine: SE ‎=0.33, SP =0.85, PPV =0.32, AUC=0.64;   Suicide attempt: Logistic regression: SE ‎=0.47, SP =0.55, PPV =0.02, AUC=0.53 K-nearest neighbors: SE ‎=0.53, SP =0.74, PPV =0.04, AUC=0.66 Decision tree: SE ‎=0.6, SP =0.83, PPV =0.07, AUC=0.77 Random forest: SE ‎=0.4, SP =0.92, PPV =0.09, AUC=0.8 Gradient boosting: SE ‎=0.47, SP =0.91, PPV =0.1, AUC=0.8 Support vector machine: SE ‎=0.4, SP =0.9, PPV =0.08, AUC=0.63 |
| Peis et al. 2019 (97) | Patients who were evaluated in psychiatric routine or psychological visits (Spain) | (1,023; N/A; N/A) | Administrative (2014-2016) | Suicidal thoughts | Cross-validation | Neural Network: AC‎=0.89, AUC=0.8329 |
| Wallace et al.‎ 2020 (98) | College students (U.S.) | (3,495; 772; 2,723);  (1,990; 408; 1,582) | Cross-sectional  Survey (2011-2017) | Suicidal thoughts | Cross-validation | Decision tree: SE ‎=0.85, SP =0.89;  Decision tree: SE ‎=0.84, SP =0.92 |
| Walsh et al.‎ 2021 (99) | Patients seen for any reason in adult inpatient, emergency department, and ambulatory surgery settings (U.S.) | (77,973; 395; 77,578);  (77,973; 85; 77,888) | Longitudinal  Administrative (2019-2020) | Suicidal thoughts | Hold-out | Random forest: AUC=0.836;  Random forest: AUC=0.797 |
| Walsh et al.‎ 2017 (100) | Adult patients with a claim code for self-injury (U.S.) | (5,167; 3,250; 1,917) | Longitudinal  Administrative ‎(N/A)‎ | Suicide attempt | Bootstrap optimism correction | Random forest: AUC=0.84 |
| Wang et al.‎ 2021 (101) | Adults recruited from the inpatient psychiatric unit (U.S.) | (85; 9; 76) | Longitudinal  Survey (2016-2018) | Suicide attempt | Cross-validation | Elastic net: AC‎=0.85, SE ‎=0.57, SP =0.88, PPV =0.39, AUC=0.89 |
| Wei et al. ‎2021 (102) | Suicide attempters (China) | (1,103; 49; 1,054) | Longitudinal  Administrative (10 years) | Suicide attempt | Cross-validation | LASSO: AUC=0.791 Random forest: AUC=0.81 Gradient boosting: AUC=0.758 Cox proportional hazard: AUC=0.779 |
| Wei & Mukherjee 2021 (103) | Students in grades 9-12 (U.S.) | (22,447; 1,631; 20,816) | Cross-sectional  Survey (1999-2017) | Suicide attempt | Hold-out | Bayesian additive regression trees: AC‎=0.8 |
| Weller et al.‎ 2021 (104) | High school students (U.S.) | (136,286; 97,927; 38,359) | Cross-sectional  Survey (2011-2017) | Suicide attempt or suicidal thoughts | Hold-out | Naive Bayes: AUC=0.52 Logistic regression: AUC=0.53 Gradient boosting: AC‎=0.91, AUC=0.91 K-nearest neighbors: AUC=0.84 |
| Zalar et al. ‎2018 (105) | Individuals registered as committed or attempted suicide (Slovenia) | (36,922; 21,913; 15,009) | Cross-sectional  Administrative (1976 - 2016) | Death by suicide | Hold-out | Super learner: AC‎=0.91 |
| Zheng et al. ‎2020 (106) | Patients with hospital visits (U.S.) | (118,252; 255; N/A) | Longitudinal Administrative (2015-2017) | Suicide attempt | Cross-validation | Deep neural network: AUC=0.769 XGBOOST: AUC=0.702 Logistic regression: AUC=0.604 |
| Zuromski et al. ‎2020 (107) | Soldiers (U.S.) | (7,677; 103; 7,574) | Longitudinal Administrative and Survey (2011-2012) | Suicide attempt | Cross-validation | Elastic net: AUC=0.755 Super learner: AUC=0.768 |
| Hong & Lee ‎2019 (108) | Depressed elderly in Korea(South Korea) | (239; 69; 170) | Cross-sectional Survey ‎(N/A)‎ | Suicidal thoughts | Cross-validation | Decision tree: AC‎=0.81, SE ‎=0.86, SP =0.68 |
| Amini et al. ‎2016(109) | Suicide attempters (Iran) | (5,414; 457; 4,957) | Cross-sectional Survey (2008-2010) | Death by suicide | Hold-out | Logistic regression: AC‎=0.65, SE ‎=0.73, SP =0.65, PPV =0.16, NPV=0.96, AUC=0.752 Neural network: AC‎=0.62, SE ‎=0.75, SP =0.6, PPV =0.15, NPV=0.96, AUC=0.748 Support vector machine: AC‎=0.67, SE ‎=0.53, SP =0.68, PPV =0.14, NPV=0.94, AUC=0.719 Decision tree: AC‎=0.49, SE ‎=0.85, SP =0.46, PPV =0.13, NPV=0.97, AUC=0.725 |
| Barak-Corren et al. 2017 (110) | Patients with inpatient and outpatient visits (U.S.) | (1,728,444; 20,246; 1,708,198);  (1,007,239; 9,068; 998,171) | Longitudinal  Administrative (1998-2012) | Death by suicide or attempt | Hold-out | Naive Bayes: AC‎=0.94, SE ‎=0.33, SP =0.95, PPV =0.06, NPV=0.99, AUC=0.77;  Naive Bayes: AC‎=0.95, SE ‎=0.34, SP =0.95, PPV =0.05, NPV=0.99, AUC=0.77 |
| García de la Garza et al. ‎2021 (111) | Non-institutionalized civilian population aged ≥ 18 (U.S.) | (34,653; 222; 34,431) | Longitudinal  Survey (2001-2005) | Suicide attempt | Cross-validation | Random forest: SE ‎=0.85, SP =0.73, AUC=0.857 |
| Karmakar et al. ‎2016 (112) | Mental health patients aged ≥ 10 who were assessed for suicide risk (Australia) | (16,858; 2,072; 14,786) | Longitudinal  Administrative (2009-2012) | Suicide attempt | Cross-validation | Linear regression: AUC=0.71 |
| Rozek et al. 2020 (113) | Military Personnel (U.S.) | (152; 76; 76) | Cross-sectional  Survey ‎(N/A)‎ | Suicide attempt or suicidal thoughts |  | Super learner: AC‎=0.87, Sensitivity ‎=0.31, SP =0.99, PPV =0.89, NPV=0.87 |
| Barros et al.‎ 2020 (114) | Patients with mood and anxiety symptomatology (Chile) | (650; 326; 324) | Cross-sectional  Survey ‎(N/A)‎ | Suicide attempt or suicidal thoughts | Cross-validation | Support vector machine: AC‎=0.78, SE ‎=0.77, SP =0.79 Decision tree: AC‎=0.67, SP =0.67, AUC=0.7335 Bayesian network: AC‎=0.71, SE ‎=0.68, SP =0.73, PPV =0.71, NPV=0.7 |
| Burke et al.‎ 2020 (115) | Emergency department and primary care patients aged 14-24 (U.S.) | (12,001; 1,113; 10,888);  (12,001; 209; 11,792) (13,325; 608; 12,717) | Cross-sectional  Survey ‎(N/A)‎ | Suicide attempt | Cross-validation and Bootstrap optimism correction | Random forest: AC‎=0.92, SE ‎=0.7, SP =0.94, AUC=0.944;  Ridge regression: AC‎=0.98, SE ‎=0.67, SP =0.99, AUC=0.984 Random forest: AC‎=0.96, SE ‎=0.7, SP =0.97, AUC=0.973 |
| Ge et al. ‎2020 (116) | Chinese patients with Major Depressive Disorder (China) | (1,916; 319; 1,597) | Longitudinal  Administrative ‎(N/A)‎ | Suicidal thoughts | Cross-validation | Neural network: AC‎=0.7, SE ‎=0.71, SP =0.67, AUC=0.76 |
| Haroz et al. 2020 (117) | Native American communities (U.S.) | (2,390; 98; 2,292) | Longitudinal  Administrative and Survey (2006 - 2017) | Suicide attempt | Cross-validation | Ridge regression: SE ‎=0.76, SP =0.76, PPV =0.13, NPV=0.99, AUC=0.87 LASSO: SE ‎=0.58, SP =0.82, PPV =0.12, NPV=0.98, AUC=0.86 Elastic net: SE ‎=0.52, SP =0.83, PPV =0.11, NPV=0.98, AUC=0.85 Logistic regression: SE ‎=0.52, SP =0.83, PPV =0.12, NPV=0.98, AUC=0.83 Decision tree: SE ‎=0.7, SP =0.81, PPV =0.13, NPV=0.98, AUC=0.81 |
| Ryan M. Hill, 2020 (118) | High- and middle-schools (U.S.) | (4,834; 192; 4,642) | Longitudinal  Survey ‎(N/A)‎ | Suicide attempt | Cross-validation | Decision tree: AC‎=0.85, SE ‎=0.7, SP =0.86;  Decision tree: AC‎=0.72, SE ‎=0.91, SP =0.71 |
| Indrawan et al. ‎2018 (119) | Patients with suicide-related behaviours and were under active treatment (Indonesia) | (2,665; 111; 2,554) | Cross-sectional  Administrative (2011-2016) | Suicide attempt or suicidal thoughts | Cross-validation | Support vector machine: AC‎=0.63 |
| Metzger et al. ‎2017 (120) | Emergency department patients (France) | (390; 98; 292) | Longitudinal  Administrative (2011-2012) | Suicide attempt | Hold-out | Random forest: SE ‎=0.96, PPV =0.93 Naive Bayes: SE ‎=0.95, PPV =0.97 Support vector machine: SE ‎=0.9, PPV =0.93 Decision tree: SE ‎=0.87, PPV =0.91 Neural network: SE ‎=0.79, PPV =0.9 Logistic regression: SE ‎=0.9, PPV =0.94 |
| Note: SE: Sensitivity; SP: Specificity; AC: Accuracy, AUC‎: Area under the receiver operating characteristic curve‎; PPV: Positive predictive value; NPV: Negative predictive value; LASSO: Least absolute shrinkage and selection operator; PTSD: Post-‎traumatic ‎stress ‎disorder; AUDIT: Alcohol ‎Use ‎Disorders ‎Identification Test; ED‎: Emergency department; N/A: Not mentioned in the study | | | | | | |

Table 4. Most important predictors of suicidal thoughts and behaviors identified in the included studies

| **Reference** | **Study population (Country)** | **Study Design Data Source (Timeframe)** | **Suicide outcomes** | **Relevant risk factors (those for the best model will be reported)** |
| --- | --- | --- | --- | --- |
| Adams et al. ‎2021 (17) | Men with incidents of substance use disorders diagnosis (Denmark);  Women with incidents of substance use disorders diagnosis (Denmark) | Longitudinal Administrative (1995-2015) | Death by suicide | Men: Those prescribed antipsychotics and diagnosed with a brief psychotic disorder; Women: Age >30, poisoning diagnosis |
| Agne et al. ‎2020 (18) | Patients with obsessive-compulsive disorder (Brazil) | Cross-sectional Administrative (2003-2009) | Suicide attempt | Previous suicide planning, previous suicide thoughts, lifetime depressive episodes, and intermittent explosive disorder |
| Bae et al. ‎2015(19) | Middle and high school students (South Korea) | Cross-sectional Survey (2011) | Suicide attempt | Intimacy with family and stress level |
| Baca-Garcia et al. ‎2007 (20) | Suicide attempters 18 years or older recruited in an emergency room (Spain) | Cross-sectional Survey (1996-1998) | Suicide attempt | Use of alcohol in intent and family history of completed suicide |
| Ballester et al. ‎2021 (21) | 18- to 24-year-old participants living in the urban area (Brazil) | Longitudinal Survey (2007-2009 and 2012-2014) | Suicide attempt or suicidal thoughts | Female, lower socioeconomic status, older age, not studying, having common mental disorder symptoms, and poor quality of life |
| Barros et al. ‎2017 (22) | Mental health patients (Chile) | Longitudinal Survey (2010-2014) | Suicide attempt or suicidal thoughts | Individual unrest, personal satisfaction, reasons for living, beliefs in one’s own capacities and coping abilities |
| Bernecker et al.‎ 2019 (23) | U.S. Army soldiers (U.S.) | Longitudinal Administrative and Survey (2011-2014) | Suicide attempt | Age, racial/ethnic minority status, education, rank, deployment history, bullying victimization, killing enemy combatants, recent jail time, and mental illness history |
| Buchman-Schmitt et al.‎ 2020 (24) | U.S. military service members (U.S.) | Longitudinal Survey (3-month follow-up) | Suicide attempt or suicidal thoughts | Age, Caucasian, having more years of military service |
| Burke et al. ‎2018 (25) | Undergraduate students (U.S.) | Cross-sectional Survey | Suicidal thoughts (suicide ideation and suicide plan); Suicide attempt | Suicidal ideation: Depression and anti-suicide function of NSSI;  Suicide plan: number of NSSI scars, anti-suicide function of NSSI, revenge function of NSSI, desire to cease NSSI, NSSI likelihood, and depression; Suicide attempt: number of NSSI scars, history of medical treatment due to NSSI, anti-suicide function of NSSI, anti-dissociation function of NSSI, and current suicide plan |
| Chen et al. ‎2020 (26) | Patients 18 to 39 years old visited psychiatric specialty care (Sweden) | Longitudinal Administrative (2011-2012) | Death by suicide or attempt | Intentional self-harm, unplanned visit to psychiatric specialty care service, diagnosis of borderline personality disorder, diagnosis of depressive disorder, recent dispensation of antidepressants and benzodiazepines, family history of suicide attempt, family history of a substance use disorder, and family history of borderline personality disorder |
| Cho et al. ‎2021 (27) | Elderly population (aged >=65 years) (South Korea) | Longitudinal Administrative (2009 - 2015) | Death by suicide | History of taking benzodiazepines, Body mass index, age, and history of taking sleeping pills |
| Cho et al. ‎2020 (28) | Health insurance subscribers and medical aid recipients who had undergone medical check-ups (South Korea) | Longitudinal Administrative (2009-2015) | Death by suicide | Strenuous exercise, alcohol use, moderate exercise, age, body mass index, γ-glutamyl transpeptidase, and walking exercise |
| Choi et al. ‎2021 (29) | Young adults (between 18 and 34 years old) (South Korea) | Cross-sectional Survey (2018-2019) | Death by suicide | Depression, anxiety, resilience, and self-esteem |
| Choi et al. ‎2018 (30) | General population (South Korea) | Longitudinal Administrative (2004-2013) | Death by suicide | Sex, age, type of insurance, household income, disability, and medical records of mental and behavioural disorders |
| Colic et al. 2018 (31) | Veterans still serving members of the Canadian Forces and Royal Canadian Mountain Police (Canada) | Cross-sectional Administrative | Suicidal thoughts | Feeling bad/like a failure/let people down, nausea/upset stomach during the last attack, taking any medication for anxiety, depression, or stress, and feeling heart race/pound. |
| Czyz et al. ‎2021 (33) | Psychiatrically hospitalized adolescents (aged 13–17) (U.S.) | Longitudinal Survey (2019-2020) | Suicidal thoughts | Ideation duration, hopelessness, burdensomeness, and self-efficacy to refrain from suicidal action |
| DelPozo-Banos et al. ‎2018 (36) | People residing in Wales at the time of their death (UK) | Longitudinal Administrative (2001-2015) | Death by suicide | Prescription of psychotropics, depression, anxiety, and self-harm |
| Fan et al. ‎2020 (37) | Patients with PTSD and bipolar disorders (U.S.) | Longitudinal Administrative (2004-2019) | Death by suicide or suicide attempt or suicidal thoughts | under antipsychotic (i.e., haloperidol) and antidepressants (i.e., citalopram and trazodone) treatment, diagnosis of autistic schizophrenic, and substance use disorder |
| Fazel et al. ‎2019 (38) | Individuals aged 15–65 with a diagnosis of severe mental illness (Sweden) | Longitudinal Administrative (2001-2008) | Death by suicide | Being hospitalized for schizophrenia-spectrum and bipolar disorders, previous self-harm, and being male |
| Gradus et al. ‎2020 (39) | General population (Denmark) | Longitudinal Administrative (2018-2019) | Death by suicide | Men: Age, physical health diagnoses, stress disorders, using antidepressants, hypnotics/sedatives, and antipsychotics; Women: Alcohol-related disorders, Prior suicide attempts, drugs used in addictive disorders, Schizophrenia, recurrent major depression, stress disorders |
| Gradus et al. ‎2017 (40) | Veterans of the Iraq and Afghanistan wars (U.S.) | Cross-sectional Survey (2009) | Suicidal thoughts | Men: depression, posttraumatic stress disorder (PTSD), and somatic complaints; Women: Sexual harassment during deployment, PTSD and depression |
| Harman et al.‎ 2021 (42) | Participant families (child and parent/caregiver dyads) (U.S.) | Longitudinal Survey | Suicidal thoughts; Suicide attempt or suicidal thoughts | Loneliness, impulsivity, feelings of being unloved, psychosis symptoms, and conduct problems |
| Hettige et al. ‎2017 (43) | Participants 18 to 75 years old and meeting schizophrenia spectrum disorders (Canada) | Cross-sectional Survey | Suicide attempt | Duration of illness, number of hospitalizations, Childhood Trauma (physical and emotional abuse), and lifetime drug abuse/ dependence |
| Hill et al. ‎2017 (44) | Adolescents (U.S.) | Longitudinal  Survey (1994-1996) | Suicidal thoughts | History of suicide ideation, depressive symptoms, suicide attempts or suicide among family and friends, social support, gender, ethnicity, hours of sleep, and school-related factors |
| Horvath et al. ‎2020 (45) | Prison population (U.S.) | Cross-sectional  Survey | Suicide attempt | Meeting five or more diagnostic criteria of borderline personality disorder (BPD) and the number of times hospitalized for psychiatric problems |
| Huang et al. ‎2020 (46) | Participants who have either engaged in NSSI and/or suicide attempt in their lifetime (Worldwide) | Longitudinal  Survey | Suicide attempt | Confidence in killing self during preparations for suicide, Intent on acting on suicide plans, lifetime history of preparation for suicide, self-rated likelihood of developing future suicide plans, and disgust with self |
| Jordan et al. ‎2018 (50) | Primary care patients (Germany) | Cross-sectional  Survey (2011-2016) | Suicidal thoughts | Feelings of depression/hopelessness, low self-esteem, worrying, and severe sleep disturbances |
| Jung et al. ‎2019 (51) | Korean adolescents (South Korea) | Cross-sectional Survey | Suicide attempt or suicidal thoughts | Sadness, violence, substance use, and stress |
| Lee et al. ‎2021 (55) | Korean adolescents (South Korea) | Cross-sectional  Survey | Suicide attempt | Experience of sadness and hopelessness, substance abuse, and violent victimization |
| Kuroki & Tilley 2012 (59) | Asian Americans (U.S.) | Cross-sectional  Survey (2002-2003) | Suicide attempt; Suicidal thoughts | Attempt: Higher levels of family conflict, low family support Perceived discrimination; Ideation: Depressive disorder, anxiety disorder, family cohesion, family conflict |
| Lee et al. ‎2019 (60) | Adolescents (South Korea) | Cross-sectional  Survey (2017) | Suicide attempt | Age, sex, breakfast consumption, experience of violence, sleep duration, perceived stress, feeling of sadness, cigarette smoking, alcohol drinking, perceived general health, perceived academic record, household economic status, and living with biological or adoptive parents |
| Lopez-Castroman et al. ‎2011(64) | Suicide attempters from Emergency Department (Spain and France) | Cross-sectional  Administrative (1994 - 2006) | Suicide attempt | Age, age at first attempt, anxiety, and educational level |
| Lyu & Zhang‎ 2019 (65) | Chinese rural residents (China) | Longitudinal  Survey (2012-2014) | Suicide attempt | Family history of suicide, mental problems, low education level, poor health, aspiration strain, hopelessness, impulsivity, and depression |
| Machado et al. ‎2021 (66) | U.S. civilian noninstitutionalized population (U.S.) | Longitudinal  Survey (3-year follow-up) | Suicide attempt | All participants: Diagnosis of borderline personality disorder, post-traumatic stress disorder, and being of Asian descent for the model in all participants;  Participants with lifetime major depressive episodes: Previous suicide attempt, borderline personality disorder, and overnight stay in hospital because of depressive symptoms |
| Mann et al. ‎2008 (68) | Patients with mood, schizophrenia spectrum, or personality disorders (U.S.) | Longitudinal  Survey (1989-1998) | Suicide attempt | Lifetime aggression, current subjective depression, suicidal ideation, and comorbid borderline personality disorder |
| Marcon et al.‎ 2019 (69) | Medical students (Brazil) | Cross-sectional  Survey | Suicide attempt | Female gender, homosexuality, low income, bullying by university peers, childhood or adult trauma, family history of suicide, past suicidal ideation, daily tobacco use, and being at severe risk for alcohol abuse |
| McKernan et al. ‎2019 (70) | Patients with Fibromyalgia (U.S.) | Longitudinal  Administrative (1998-2017) | Suicide attempt; Suicidal thoughts | Suicide attempt: Drug dependence, obesity, mental illness, and inpatient utilization; Suicidal thoughts: Polysomatic symptoms (fatigue, dizziness, weakness), serious and persistent mental illness and inpatient utilization |
| Modai et al.‎1998 (71) | Hospitalized psychiatric patients (Israel) | Longitudinal  Administrative | Suicide attempt | Subjective feelings of helplessness and hopelessness |
| Miché et al.‎ 2020 (72) | Adolescents and young adults aged 14–24 years (Germany) | Longitudinal  Survey (1995-2005) | Suicide attempt | Prior suicide attempts, prior help-seeking, and number of lifetime mental disorders |
| Modai et al. ‎1999(73) | Psychiatric patients (Israel) | Longitudinal  Survey | Suicide attempt | Living alone, compliance to treatment, drug abuse or dependence, functional level, non-paranoid delusions, and suicide of one degree relative |
| Na et al. ‎2022 (75) | Community-dwelling elderly aged of >55 years (South Korea) | Longitudinal  Survey | Suicidal thoughts | Depression, satisfaction with leisure life, health satisfaction, satisfaction with family relationships, and self-esteem |
| Naghavi et al. 2020 (76) | University students (Iran) | Cross-sectional  Survey (March 2020 and May 2020) | Suicide attempt or suicidal thoughts | Exposure to trauma, history of psychological illness, PTSD symptoms, positive mental health, depression symptoms, post-traumatic growth, and social support |
| Parghi et al. ‎2020 (80) | High‐risk psychiatric inpatients (U.S.) | Longitudinal  Survey (2016-2019) | Suicide attempt | Affective dyscontrol (“Felt unusual physical sensations that you have never felt before,” “Felt the blood rushing through your veins,” “Became afraid that you would die,”), loss of cognitive control (“Felt your head could explode from too many thoughts”), and entrapment/frantic hopelessness and Emotional pain (“Felt urge to escape the pain was very hard to control”) |
| Passos et al. ‎2016 (81) | Patients with mood disorders (U.S.) | Cross-sectional  Survey (2006-2010) | Suicide attempt | Previous hospitalizations for depression, history of psychosis, cocaine dependence, and post-traumatic stress disorder (PTSD) comorbidity |
| Rosellini et al. 2017 (82) | New U.S. Army soldiers (U.S.) | Cross-sectional  Survey (2011-2012) | Suicide attempt | Female, anxiety disorders, total number of lifetime disorders, lifetime treatment of mental disorders, family history of mental illness, and non-secure attachment styles |
| Rosellini et al. ‎2018 (83) | U.S. Army soldiers (U.S.) | Longitudinal  Survey (2012–2013) | Suicidal thoughts | Mental disorders (major depressive episode, generalized anxiety disorder), suicidality, anger attacks and violence during deployment (got into a fight, bullied/hazed) |
| Ryu et al. ‎2019 (84) | Individuals aged over 19 years in Korea (South Korea) | Longitudinal  Survey (19 years) | Suicide attempt | Days of feeling sick or in discomfort, Alcohol Use Disorders Identification Test (AUDIT) score, amount of daily smoking, average work week, household composition, EuroQoL-Visual Analogue Scale (VAS), age, frequency of drinking, number of household members, and depressed mood over two weeks |
| Ryu et al. ‎2018 (85) | General Population (South Korea) | Longitudinal  Survey (19 years) | Suicidal thoughts | Depression, anxiety, and stress |
| Shen et al. ‎2020 (90) | Chinese medical college students (China) | Cross-sectional  Survey (between January and March of 2018) | Suicide attempt | Suicidal ideation, suicide plan, anxiety, depression, relationship with father, alcohol consumption, physical disorder history, family income, family type, and relationship with mother |
| Simon et al.‎ 2018 (91) | Patients age 13 or older who made specialty mental health visits and primary care visits with mental health diagnoses (U.S.) | Longitudinal  Administrative (2009-2015) | Suicide attempt; Death by suicide | Attempt: Prior suicide attempt, mental health and substance use diagnoses, having thoughts of death or self-harm nearly every day, prior inpatient or emergency mental health care; Death by suicide: Prior suicide attempt, mental health and substance use diagnoses, having thoughts of death or self-harm nearly every day, and prior inpatient or emergency mental health care |
| Stanley et al. ‎2022 (93) | U.S. Army soldiers after leaving active duty (U.S.) | Longitudinal  Survey (2016–2018, 2018–2019) | Suicide attempt | Self-reported lifetime suicide plan |
| Tran et al. ‎2014 (94) | Patients aged 10 years or over, and had received at least one suicide risk assessment (Australia) | Longitudinal  Administrative | Suicide attempt | Having an ED visit or inpatient admission with a high-lethality diagnosis (e.g., severe injuries and poisoning or very high alcohol level in blood), injuries and poisoning, being male with mental health problems who moved home, prior emergency visits, addictive drugs treatment, and having an ED visit or inpatient admission with a moderate-lethality diagnosis (e.g., moderate severity of poisoning or high alcohol level in blood, or personal history of self-harm) |
| van Mens et al. ‎2020 (96) | Young adults (18–34-year old’s) across Scotland (Scotland) | Longitudinal  Survey (one year follow up) | Suicidal thoughts; Suicide attempt | Suicidal thoughts: Internal entrapment, defeat and perceived burdensomeness; Suicide attempt: Internal entrapment and depressive symptoms |
| Peis et al. 2019 (97) | Patients who were evaluated in psychiatric routine or psychological visits (Spain) | Administrative (2014-2016) | Suicidal thoughts | Thought about self-harm, previous suicidal thoughts, previous suicide attempts, unemployed with benefits, living with siblings, shared residence, mental disorders due to known physiological conditions, electroconvulsive therapy, living with offspring, temporary disability |
| Walsh et al. ‎2017 (100) | Adult patients with a claim code for self-injury (U.S.) | Longitudinal  Administrative | Suicide attempt | Age, poison with psychotropic agent, Body Mass Index, number of outpatient visits preceding 1 year, number of Inpatient visits preceding 1 year, and number of emergency room visits preceding 1 year |
| Wang et al. ‎2021 (101) | Adults recruited from the inpatient psychiatric unit (U.S.) | Longitudinal  Survey (2016-2018) | Suicide attempt | Probability of acute change in the desire, intention, and ability to resist the urge to kill oneself |
| Wei et al. ‎2021 (102) | Suicide attempters (China) | Longitudinal  Administrative (10 years) | Suicide attempt | Mental disorder, history of suicide attempts, age, suicide intent, marital status |
| Wei & Mukherjee ‎2021 (103) | Students in grades 9-12 (U.S.) | Cross-sectional  Survey (1999-2017) | Suicide attempt | Being sad/hopeless, having safety concerns at school, physical fighting, inhalant usage, illegal drug consumption at school, current cigarette usage, having first sex at an early age (below 15 years of age), being in the minority groups (American Indian/Alaska Natives, Hispanics/Latinos), and being a female |
| Weller et al. ‎2021 (104) | High school students (U.S.) | Cross-sectional  Survey (2011-2017) | Suicide attempt or suicidal thoughts | Familial life (being in a family where there are serious arguments or that yells and insults each other), age at first alcohol use, demographics (age, gender), and peer acceptance at school (being threatened or harassed through digital media or being picked on or bullied at school in the past 12 months) |
| Zheng et al.‎ 2020 (106) | Patients with hospital visits (U.S.) | Longitudinal Administrative (2015-2017) | Suicide attempt | Age, mental health conditions or pain, previous suicide attempts, psychotropic medication treatment, having open wounds or injuries due to unspecific reasons |
| Hong & Lee 2019 (108) | Depressed elderly in Korea (South Korea) | Cross-sectional Survey | Suicidal thoughts | Perceived stress level, household income level, quality of life and restriction of activity |
| Barak-Corren et al.‎ 2017 (110) | Patients with inpatient and outpatient visits (U.S.) | Longitudinal  Administrative (1998-2012) | Death by suicide or attempt | Substance-related disorders, personality disorders, alcohol-related disorders, schizophrenia and other psychotic disorders, open wounds, and superficial injury |
| García de la Garza et al. ‎2021 (111) | Non-institutionalized civilian population 18 years and older (U.S.) | Longitudinal  Survey (2001-2005) | Suicide attempt | Feeling downhearted, doing activities less carefully or accomplishing less because of emotional problems, younger age, lower educational achievement, and recent financial hardship |
| Karmakar et al. ‎2016 (112) | Mental health patients who aged >10 and underwent assessment for suicide risk (Australia) | Longitudinal  Administrative (2009-2012) | Suicide attempt | History of physical illnesses (ICD-10 codes, without chapter V, Mental and behavioral disorders) |
| Burke et al.‎ 2020 (115) | Emergency department and primary care patients aged 14-24 (U.S.) | Cross-sectional  Survey | Suicide attempt | History of active and passive suicidal ideation, suicide planning, and non-suicidal self-injury |
| Ge et al. ‎2020 (116) | Chinese patients with Major Depressive Disorder (China) | Longitudinal  Administrative | Suicidal thoughts | Low free triiodothyronine (FT3), low free thyroxine (FT4), severity of depressive symptoms and work status |
| Haroz et al. ‎2020 (117) | Native American communities (U.S.) | Longitudinal  Administrative and Survey (2006 - 2017) | Suicide attempt | High school degree, history of domestic violence, knowing someone who attempted suicide in last 6 months, using marijuana in last 2 weeks, completed middle school, binge substance use in last 2 weeks, female, and previous suicide attempt |
| Hill et al. ‎2020 (118) | Adolescents (U.S.) | Longitudinal  Survey | Suicide attempt | Suicide ideation, frequency ‎of feeling tired for no ‎reason, perceived chance of ‎getting a sexually ‎transmitted disease, running ‎away from home, Asian or ‎Pacific Islander ethnicity, and having a friend die by ‎suicide in the previous year‎ |
| Indrawan et al. ‎2018 (119) | Patients with suicide-related behaviours and were under active treatment (Indonesia) | Cross-sectional  Administrative (2011-2016) | Suicide attempt or suicidal thoughts | Disease diagnosis, profession, education, payment type/health insurance type, domicile, and age |
| PTSD: Post-‎traumatic ‎stress ‎disorder; AUDIT: Alcohol ‎Use ‎Disorders ‎Identification Test; ED‎: Emergency department; The following studies did not report the risk factors included in the final model (risk factors were unclear (even in the article) or not explicitly mentioned as the most important risk factors):(32, 34, 35, 41, 47-49, 52-54, 56-58, 61-63, 67, 74, 77-79, 86-89, 92, 95, 98, 99, 105, 107, 109, 113, 114, 120) | | | | |
