# Supplementary material for: The use of machine learning on administrative and survey data to predict suicidal thoughts and behaviors: a systematic review
Source: Front Psychiatry. 2024 Mar 4;15:1291362. doi: 10.3389/fpsyt.2024.1291362 (PMC10944962; doi:10.3389/fpsyt.2024.1291362)
Supplement: Supplementary file 2 [file Table_2.docx]

Appendices

*Appendix 1*

**PubMed**

| Search | PubMed Query 11 May 2022 | Items found |
| --- | --- | --- |
| #1 | "Suicide"[MeSH Terms] OR "Suicidal Ideation"[MeSH Terms] OR "suicide, attempted"[MeSH Terms] OR "suicide, completed"[MeSH Terms] OR "Self-Injurious Behavior"[MeSH Terms] | 79,295 |
| #2 | "suicid*"[Title/Abstract] OR "death by suicide"[Title/Abstract] OR "attempt* suicide*"[Title/Abstract] OR "self-injur*"[Title/Abstract] | 92,609 |
| #3 | #1 OR #2 | 115,258 |
| #4 | "Machine Learning"[MeSH Terms] OR "Deep Learning"[MeSH Terms] OR "Supervised Machine Learning"[MeSH Terms] OR "Support Vector Machine"[MeSH Terms] OR "Unsupervised Machine Learning"[MeSH Terms] OR "Artificial Intelligence"[MeSH Terms] OR "Computer Heuristics"[MeSH Terms] OR "neural networks, computer"[MeSH Terms] OR "Algorithms"[MeSH Terms] OR "Mathematical Concepts"[MeSH Terms] OR "Markov Chains"[MeSH Terms] OR "Decision Trees"[MeSH Terms] OR "Computing Methodologies"[MeSH Terms] OR "decision support systems, clinical"[MeSH Terms] OR "decision making, computer assisted"[MeSH Terms] OR "Decision Support Techniques"[MeSH Terms] OR "Data Mining"[MeSH Terms] OR "Big Data"[MeSH Terms] OR "Least-Squares Analysis"[MeSH Terms] | 1,917,566 |
| #5 | "machine learning"[Title/Abstract] OR "deep learning"[Title/Abstract] OR "unsupervised machine ‎learning"[Title/Abstract] OR "computer reasoning"[Title/Abstract] OR "boosting"[Title/Abstract] OR "naive bay*"[Title/Abstract] OR "stepwise regression"[Title/Abstract] OR "q learning"[Title/Abstract] OR "Markov"[Title/Abstract] OR "reinforcement"[Title/Abstract] OR "k nearest neighbor*"[Title/Abstract] OR "k mean cluster*"[Title/Abstract] OR "polynomial regression"[Title/Abstract] OR "regression tree*"[Title/Abstract] OR "bayesian"[Title/Abstract] OR "random forest"[Title/Abstract] OR "support vector machine"[Title/Abstract] OR "machine intelligence"[Title/Abstract] OR "comput* intelligence*"[Title/Abstract] OR "neural network*"[Title/Abstract] OR "decision technique*"[Title/Abstract] OR "decision system*"[Title/Abstract] OR "decision tree*"[Title/Abstract] OR "artificial intelligence"[Title/Abstract] OR "algorithm*"[Title/Abstract] OR "mathematical concepts"[Title/Abstract] OR "supervised machine learning"[Title/Abstract] OR "exploratory"[Title/Abstract] OR "statistical learning"[Title/Abstract] OR "big data"[Title/Abstract] OR "data mining*"[Title/Abstract] OR "Xgboost"[Title/Abstract] OR "adaboost"[Title/Abstract] OR "least square*"[Title/Abstract] | 711,812 |
| #6 | #4 OR #5 | 2,352,554 |
| #7 | #3 AND #6 | 8,536 |
| #8 | "social media"[Title/Abstract] OR "Twitter"[Title/Abstract] OR "Instagram"[Title/Abstract] OR "Facebook"[Title/Abstract] OR "YouTube"[Title/Abstract] | 27,513 |
| #13 | #7 NOT #8 | 8,226 |

**Ovid Medline**

| Search | Medline Query 11 May 2022 | Items found |
| --- | --- | --- |
| 1 | suicide/ or suicidal ideation/ or suicide, attempted/ or suicide, completed/ | ‎65182‎ |
| 2 | self-injurious behavior/ or suicide/ | 50893 |
| 3 | suicid*.tw,kf. | ‎89393‎ |
| 4 | "attempt* suicide*‎".tw,kf. | 7212 |
| 5 | ("death by suicide‎" or "self-injur*"‎).tw,kf. | ‎6479‎ |
| 6 | 1 or 2 or 3 or 4 or 5 | 110024 |
| 7 | artificial intelligence/ or machine learning/ or deep learning/ or supervised machine learning/ or unsupervised machine learning/ | ‎66100‎ |
| 8 | algorithms/ or computer heuristics/ or neural networks, computer/ | 313166 |
| 9 | Support Vector Machine/ | ‎9118‎ |
| 10 | mathematical concepts/ | 4928 |
| 11 | data mining/ | ‎10188‎ |
| 12 | Big Data/ | 2198 |
| 13 | computing methodologies/ | ‎1226‎ |
| 14 | Decision Trees/ or least-squares analysis/ or markov chains/‎ | 42598 |
| 15 | decision support techniques/ | ‎22195‎ |
| 16 | Decision Support Systems, Clinical/ | 9066 |
| 17 | decision making, computer-assisted/ | ‎2873‎ |
| 18 | 7 or 8 or 9 or 10 or 11 or 12 or 13 or 14 or 15 or 16 or 17 | 423590 |
| 19 | ("machine learning" or "deep learning" or "‎supervised ‎machine learning" or "un‎supervised ‎machine learning" or "mathematical concepts" or "‎algorithm*" or "artificial intelligence‎").tw,kf. | ‎380971‎ |
| 20 | ("decision tree*" or "decision* system*" or "decision technique*" or "neural network*").tw,kf. | 87458 |
| 21 | ("comput* intelligence*" or "machine intelligence" or "support vector machine").tw,kf. | ‎17321‎ |
| 22 | ("random forest" or "bayesian" or "regression tree*" or "polynomial regression" or "k#mean cluster*" or "k#nearest neighbor*" or "reinforcement").tw,kf. | 118308 |
| 23 | ("markov" or "q#learning" or "stepwise regression" or "na#ve bay*" or "boosting" or "computer reasoning" or "data mining*").tw,kf. | ‎66688‎ |
| 24 | ("big data" or "statistical learning" or "exploratory" or "xgboost" or "adaboost" or "least square*").tw,kf. | 151006 |
| 25 | 19 or 20 or 21 or 22 or 23 or 24 | ‎712625‎ |
| 26 | 18 or 25 | 911109 |
| 27 | 6 and 26 | ‎2865‎ |
| 28 | (“social media” OR Twitter OR Instagram OR ‎Facebook OR YouTube).tw,kf | 28145 |
| 29 | (6 and 26) not 28 | ‎2765‎ |

**Embase**

| Search | Embase Query 11 May 2022 | Items found |
| --- | --- | --- |
| 1 | exp suicide/ | ‎66067‎ |
| 2 | suicide attempt/ | 36826 |
| 3 | suicidal ideation/ | ‎26644‎ |
| 4 | suicid*.tw,kf. | 116738 |
| 5 | "attempt* suicide*‎".tw,kf. | ‎9784‎ |
| 6 | ("death by suicide‎" or "self-injur*").tw,kf. | 8217 |
| 7 | 1 or 2 or 3 or 4 or 5 or 6 | ‎150745‎ |
| 8 | exp artificial intelligence/ | ‎60512‎ |
| 9 | exp machine learning/ or artificial neural network/ or computer heuristics/ or data mining/ or supervised machine learning/ or support vector machine/ or unsupervised machine learning/ or algorithm/ | 578120 |
| 10 | exp deep learning/ | ‎24090‎ |
| 11 | exp big data/ | 4278 |
| 12 | exp Markov chain/ or least square analysis/ | ‎22847‎ |
| 13 | exp "decision tree"/ | 17307 |
| 14 | clinical decision support system/ | ‎4594‎ |
| 15 | ("machine learning" or "deep learning" or "supervised ‎machine learning" or "unsupervised ‎machine learning" or "mathematical concepts" or "‎algorithm*" or "artificial intelligence‎").tw,kf. | 476882 |
| 16 | ("decision tree*" or "decision system*" or "decision technique*" or "neural network*").tw,kf. | ‎108228‎ |
| 17 | ("comput* intelligence*" or "machine intelligence" or "support vector machine").tw,kf. | 21370 |
| 18 | ("random forest" or "bayesian" or "regression tree*" or "polynomial regression" or "k#mean cluster*" or "k#nearest neighbor*" or "reinforcement").tw,kf. | ‎137994‎ |
| 19 | ("markov" or "q#learning" or "stepwise regression" or "na#ve bay*" or "boosting" or "computer reasoning" or "data mining*").tw,kf. | 85339 |
| 20 | ("big data" or "statistical learning*" or "exploratory" or "xgboost" or "adaboost" or "least square*").tw,kf. | ‎196132‎ |
| 21 | 7 or 8 or 9 or 10 or 11 or 12 or 13 or 14 or 15 or 16 or 17 or 18 or 19 | 1111971 |
| 22 | ("social media" or Twitter or Instagram or Facebook or YouTube).tw,kf. | 35762 |
| 23 | 7 and 21 | ‎3970‎ |
| 24 | (7 and 21) not 22 | ‎3858‎ |

**‎PsycINFO**

| Search | PsycINFO Query 11 May 2022 | Items found |
| --- | --- | --- |
| 1 | exp suicide/ or attempted suicide/ or self-injurious behavior/ or suicidal ideation/ | ‎46742‎ |
| 2 | suicid*.tw,id. | 72950 |
| 3 | "attempt* suicide*‎".tw,id. | ‎6861‎ |
| 4 | ("death by suicide‎" or self-injur*).tw,id. | 8047 |
| 5 | 1 or 2 or 3 or 4 | ‎78369‎ |
| 6 | exp artificial intelligence/ | 26407 |
| 7 | machine learning/ or neural networks/ | ‎26140‎ |
| 8 | heuristics/ or decision support systems/ | 7798 |
| 9 | algorithms/ or "mathematics (concepts)"/ | ‎26044‎ |
| 10 | data mining/ or big data/ | 4030 |
| 11 | exp Least Squares/ exp Markov Chains/ | ‎2915‎ |
| 12 | ("machine learning" or "deep learning" or "‎supervised‎ ‎machine learning" or "un‎supervised‎ ‎machine learning" or "mathematical concepts" or "‎algorithm*" or "artificial intelligence‎").tw,id. | 50745 |
| 13 | ("decision tree*" or "decision system*" or "decision technique*" or "neural network*").tw,id. | ‎23540‎ |
| 14 | ("comput* intelligence*" or "machine intelligence" or "support vector machine").tw,id. | 2699 |
| 15 | ("random forest" or "bayesian" or "regression tree*" or "polynomial regression" or "k#mean cluster*" or "k#nearest neighbor*" or "reinforcement").tw,id. | ‎60545‎ |
| 16 | ("markov" or "q#learning" or "stepwise regression" or "na#ve bay*" or "boosting" or "computer reasoning" or "data mining*").tw,id. | 13696 |
| 17 | ("big data" or "statistical learning" or "exploratory" or "xgboost" or "adaboost" or "least square*").tw,id. | ‎84382‎ |
| 18 | 6 or 7 or 8 or 9 or 10 or 11 or 12 or 13 or 14 or 15 or 16 or 17 | 238587 |
| 19 | ("social media" or Twitter or Instagram or Facebook or YouTube).tw,id. | ‎23294‎ |
| 20 | 5 and 18 | 2478 |
| 21 | (5 and 18) not 19 | 2429 |

**Web of Science**

| Search | Web of Science Query ‎11 May 2022‎ | Items found |
| --- | --- | --- |
| 1 | ((TI=(‎"suicid*" OR ‎ "attempt* suicide*" OR "death by ‎suicide" ‎ OR "self-injur*‎" OR ‎ ‎‎"suicidal ‎ideation" ‎ OR ‎‎ "suicide ‎attempted" OR "suicide completed"‎)) OR AB=(‎"suicid*" OR ‎ "attempt* suicide*" OR "death by ‎suicide" ‎ OR "self-injur*‎" OR ‎ ‎‎"suicidal ‎ideation" ‎ OR ‎‎ "suicide ‎attempted" OR "suicide completed"‎)) OR AK=(‎"suicid*" OR ‎ "attempt* suicide*" OR "death by ‎suicide" ‎ OR "self-injur*‎" OR ‎ ‎‎"suicidal ‎ideation" ‎ OR ‎‎ "suicide ‎attempted" OR "suicide completed"‎) | [109,462](https://www-webofscience-com.myaccess.library.utoronto.ca/wos/woscc/summary/d2aacc63-f4bc-4b33-8fdb-8e680f1713d6-3727f40a/relevance/1) |
| 2 | ((TI=("machine learning" or "deep learning" or "supervised ‎machine learning" or "unsupervised ‎machine learning" or ‎‎ ‎‎"mathematical concepts" ‎‎or "‎algorithm*" or "artificial intelligence‎" ‎ or ‎ ‎‎"decision tree*" or "decision system*" or ‎‎ ‎‎"decision technique*" or "neural ‎‎network*" or "comput* intelligence*" or "machine intelligence" ‎‎or "support ‎vector machine" or "random forest" or "bayesian" or "regression tree*" or ‎‎ ‎‎‎"polynomial regression" or ‎‎ "k-mean cluster*" or "k-nearest neighbor*" or ‎‎"reinforcement" or ‎‎ ‎‎"markov" or "q-learning" or "stepwise regression" or "naive ‎bay*" ‎‎or "boosting" or "computer reasoning" ‎or "data mining*" or "big data" or "statistical learning" ‎or ‎‎ "exploratory" or ‎‎"xgboost" or "adaboost" or "least square*")) OR AB=("machine learning" or "deep learning" or "supervised ‎machine learning" or "unsupervised ‎machine learning" or ‎‎ ‎‎"mathematical concepts" ‎‎or "‎algorithm*" or "artificial intelligence‎" ‎ or ‎ ‎‎"decision tree*" or "decision system*" or ‎‎ ‎‎"decision technique*" or "neural ‎‎network*" or "comput* intelligence*" or "machine intelligence" ‎‎or "support ‎vector machine" or "random forest" or "bayesian" or "regression tree*" or ‎‎ ‎‎‎"polynomial regression" or ‎‎ "k-mean cluster*" or "k-nearest neighbor*" or ‎‎"reinforcement" or ‎‎ ‎‎"markov" or "q-learning" or "stepwise regression" or "naive ‎bay*" ‎‎or "boosting" or "computer reasoning" ‎or "data mining*" or "big data" or "statistical learning" ‎or ‎‎ "exploratory" or ‎‎"xgboost" or "adaboost" or "least square*")) OR AK=("machine learning" or "deep learning" or "supervised ‎machine learning" or "unsupervised ‎machine learning" or ‎‎ ‎‎"mathematical concepts" ‎‎or "‎algorithm*" or "artificial intelligence‎" ‎ or ‎ ‎‎"decision tree*" or "decision system*" or ‎‎ ‎‎"decision technique*" or "neural ‎‎network*" or "comput* intelligence*" or "machine intelligence" ‎‎or "support ‎vector machine" or "random forest" or "bayesian" or "regression tree*" or ‎‎ ‎‎‎"polynomial regression" or ‎‎ "k-mean cluster*" or "k-nearest neighbor*" or ‎‎"reinforcement" or ‎‎ ‎‎"markov" or "q-learning" or "stepwise regression" or "naive ‎bay*" ‎‎or "boosting" or "computer reasoning" ‎or "data mining*" or "big data" or "statistical learning" ‎or ‎‎ "exploratory" or ‎‎"xgboost" or "adaboost" or "least square*") | [3,635,919](https://www-webofscience-com.myaccess.library.utoronto.ca/wos/woscc/summary/2870f89a-6b61-4411-97e5-b90e4766268e-372930d3/relevance/1) |
| 3 | ((TI=("social media" or Twitter or Instagram or Facebook or YouTube)) OR AB=("social media" or Twitter or Instagram or Facebook or YouTube)) OR AK=("social media" or Twitter or Instagram or Facebook or YouTube) | [118,923](https://www-webofscience-com.myaccess.library.utoronto.ca/wos/woscc/summary/1e303bc6-c047-4cd0-b250-39c1e0af4eeb-372944a2/relevance/1) |
| 4 | #1 AND #2 | [3,024](https://www-webofscience-com.myaccess.library.utoronto.ca/wos/woscc/summary/e69f2f06-8dc9-4a5f-9755-b68d143c8d31-37294c22/relevance/1) |
| 5 | #4 NOT #3 | [2,841](https://www-webofscience-com.myaccess.library.utoronto.ca/wos/woscc/summary/ed8bfeb5-fa99-49db-bd30-fdb54ad3b97f-375317fe/relevance/1) |

**CINAHL**

| Search | CINAHL Query ‎11 May 2022‎ | Items found |
| --- | --- | --- |
| 1 | (MH "Suicidal Ideation") OR (MH "Suicide, Attempted") OR (MH "Suicide") | 32,390 |
| 2 | (MH "Self-Injurious Behavior") | 4,781 |
| 3 | TI ‎"suicid*" OR ‎ "attempt* suicide*" OR "death by ‎suicide" ‎ OR "self-injur*‎" | 29,378 |
| 4 | AB ‎"suicid*" OR ‎ "attempt* suicide*" OR "death by ‎suicide" ‎ OR "self-injur*‎" | 33,327 |
| 5 | S1 OR S2 OR S3 OR S4 | 48,974 |
| 6 | (MH "Decision Support Systems, Clinical") | 6,101 |
| 7 | (MH "Decision Trees") OR (MH "Decision Support Techniques") | 10,093 |
| 8 | (MH "Computing Methodologies") OR (MH "Neural Networks (Computer)") OR (MH "Machine Learning") OR (MH "Support Vector Machine") OR (MH "Deep Learning") OR (MH "Artificial Intelligence") OR (MH "Decision Making, Computer Assisted") OR (MH "Algorithms") | 53,066 |
| 9 | TI ‎"machine learning" or "deep learning" or "supervised‎ machine learning" or ‎‎ "unsupervised‎ machine learning" ‎‎OR "mathematical concepts" ‎or "‎algorithm*" or "artificial intelligence‎" ‎ or ‎ ‎‎"decision tree*" or "decision system*" or ‎‎ "decision technique*" or "neural ‎‎network*" or "comput* intelligence*" or "machine intelligence" ‎or "support ‎vector machine" or "random forest" or "bayesian" or "regression tree*" or ‎‎ ‎‎"polynomial regression" or ‎‎ "k-mean* cluster*" or "k-nearest neighbor*" or ‎‎"reinforcement" or ‎‎ "markov" or "q-learning" or "stepwise regression" or "naive ‎bay*" ‎or "boosting*" or "computer reasoning" ‎or "data mining*" or "big data" or "statistical learning" or ‎‎ "exploratory" or ‎‎"xgboost" or "adaboost" or ‎‎"least square*" | 131,939 |
| 10 | AB ‎"machine learning" or "deep learning" or "supervised‎ machine learning" or ‎‎ "unsupervised‎ machine learning" ‎‎OR "mathematical concepts" ‎or "‎algorithm*" or "artificial intelligence‎" ‎ or ‎ ‎‎"decision tree*" or "decision system*" or ‎‎ "decision technique*" or "neural ‎‎network*" or "comput* intelligence*" or "machine intelligence" ‎or "support ‎vector machine" or "random forest" or "bayesian" or "regression tree*" or ‎‎ ‎‎"polynomial regression" or ‎‎ "k-mean* cluster*" or "k-nearest neighbor*" or ‎‎"reinforcement" or ‎‎ "markov" or "q-learning" or "stepwise regression" or "naive ‎bay*" ‎or "boosting*" or "computer reasoning" ‎or "data mining*" or "big data" or "statistical learning" or ‎‎ "exploratory" or ‎‎"xgboost" or "adaboost" or ‎‎"least square*"‎ | 132,949 |
| 11 | S6 OR S7 OR S8 OR S9 OR S10 | 183,195 |
| 12 | TI "social media" or Twitter or Instagram or Facebook or YouTube‎ | 15,734 |
| 13 | AB "social media" or Twitter or Instagram or Facebook or YouTube‎ | 18,952 |
| 14 | S12 OR S13 | 21,945 |
| 15 | S5 AND S11 | 1,459 |
| 16 | S15 NOT S14 | 1,422 |

**AMED (Allied and Complementary Medicine)**

| Search | AMED Query ‎11 May 2022‎ | Items found |
| --- | --- | --- |
| 1 | suicide/ or suicide attempted/ | 565 |
| 2 | self injurious behavior/ | 216 |
| 3 | "suicid*‎".ti,ab. | 1368 |
| 4 | "‎attempt* suicide*".ti,ab. | 48 |
| 5 | "death by suicide‎".ti,ab. | 16 |
| 6 | "self-injur*".ti,ab. | 235 |
| 7 | 1 or 2 or 3 or 4 or 5 or 6 | ‎1716‎ |
| 8 | algorithms/ | 301 |
| 9 | ("machine learning" or "deep learning" or "supervised‎ machine learning" or "unsupervised ‎machine ‎learning").ti,ab. | 83 |
| 10 | ("mathematical concepts" or "‎algorithm*" or "artificial intelligence" or "decision ‎tree*" or "decision system*" or "decision ‎technique*" or "neural network*").ti,ab. | 1366 |
| 11 | ("comput* ‎intelligence*" or "machine intelligence" or "support vector machine" or "random forest" or "bayesian" or "regression tree*").ti,ab. | 171 |
| 12 | ("polynomial ‎regression" or "k-mean cluster*" or "k-nearest ‎neighbor*" or "reinforcement" or "Markov" or "Q-‎learning" or "tepwise regression").ti,ab. | 438 |
| 13 | ("naive bay*" or "boosting" or "computer reasoning" or "data mining*" or "big data" or "statistical learning" or "exploratory" or "xgboost" or "adaboost" or "least square").ti,ab. | 2046 |
| 14 | 8 or 9 or 10 or 11 or 12 or 13 | 4006 |
| 15 | 7 and 14 | 41 |
| 16 | ‎("social media" or Twitter or Instagram or Facebook or YouTube).ti,ab.‎ | 158 |
| 17 | (7 and 14) not 16 | 41 |

*Appendix 2*

| N | Author, Date, Country | Reasons for exclusions |
| --- | --- | --- |
| 1. | Alarca˜o et al. 2020, Brazil[1] | Not at the individual level to decide to commit suicide. |
| 2. | Choo et al., 2019, ‎Singapore[2] | Not using machine learning ‎to predict ‎‎suicide, suicide ‎ideation, or behavior. ‎‎ |
| 3. | Yin et al., 2021, China‎[3] | This study mixed self-harm with suicide |
| 4. | Allen et al., 2019, U.S.‎[4] | This is a review study. |
| 5. | Batterham et al., 2012, Australia[5] | Not using machine learning to predict suicide, suicide ideation, or behavior. The authors assessed the association between suicide and other variables. |
| 6. | Bayramli et al., 2022, U.S.[6] | This study applied natural language processing (NLP) concept to detect patients with suicide attempts |
| 7. | Bossarte et al., 2021, U.S.[7] | This is a commentary article |
| 8. | Brown et al., 2020, U.S.[8] | Not at the individual level to decide to ‎commit suicide.‎ |
| 9. | Zubizarreta et al., 2022, U.S.‎[9] | Not using machine learning to predict ‎‎‎‎‎‎‎‎suicide, suicide ideation, or behavior. ‎‎‎‎‎‎‎‎The authors assessed the association ‎‎‎‎‎‎‎‎between suicide and other variables.‎ |
| 10. | Chekroud, 2018, U.S.[10] | This article was an editorial. |
| 11. | Cho, 2020, South Korea[11] | Not using machine learning to predict suicide, suicide ideation, or behavior. The authors assessed the association between suicide and other variables. |
| 12. | Choo et al., 2014, Singapore[12] | Not using machine learning to predict ‎suicide, suicide ideation, or behavior. ‎The authors assessed the association ‎between suicide and other variables.‎ |
| 13. | Ilan Modai et al., ‎2004,‎ ‎Israel‎[13] | This article was a brief report. ‎ |
| 14. | Pestian et al., 2017, U.S.‎[14] | Not using administrative data ‎ |
| 15. | Connolly et al., 2017, U.S.[15] | Not at the individual level to decide to ‎commit suicide.‎ |
| 16. | Desjardins et al., 2016, Canada[16] | Not at the individual level to decide to ‎commit suicide.‎ |
| 17. | Edgcomb et al., 2020, U.S.[17] | Not at the individual level to decide to ‎commit suicide.‎ |
| 18. | Edgcomb et al., 2021, U.S.[18] | Not at the individual level to decide to ‎commit suicide.‎ |
| 19. | Mezuk et al., 2019, U.S.‎[19] | Not using machine learning to predict ‎‎‎‎‎‎‎suicide, suicide ideation, or behavior. ‎‎‎‎‎‎‎The authors assessed the association ‎‎‎‎‎‎‎between suicide and other variables.‎ |
| 20. | García-Martín et al., 2020, Spain[20] | Not using machine learning to predict ‎suicide, suicide ideation, or behavior. ‎The authors assessed the association ‎between suicide and other variables.‎ |
| 21. | Gradus et al., 2021, U.S.[21] | This article was a commentary |
| 22. | Hwang et al., 2020, Canada[22] | This article was a letter to the editor |
| 23 | Ibala et al., 2021, Nigeria, Uganda, and Malawi[23] | Not using machine learning to predict ‎suicide, suicide ideation, or behavior. ‎The authors assessed the association ‎between suicide and other variables.‎ |
| 24. | Ilgen et al., 2009, U.S.[24] | Not using machine learning to predict ‎suicide, suicide ideation, or behavior. ‎The authors assessed the association ‎between suicide and other variables.‎ |
| 25. | Jang et al., 2020, South Korea[25] | Not using machine learning to predict ‎suicide, suicide ideation, or behavior. ‎The authors assessed the association ‎between suicide and other variables.‎ |
| 26. | Jiang et al., 2021, Denmark[26] | This manuscript was a dissertation |
| 27. | Kan et al., 2022, China[27] | Not using machine learning to predict ‎suicide, suicide ideation, or behavior. ‎The authors assessed the association ‎between suicide and other variables.‎ |
| 28. | Kang et al., 2015, U.S.[28] | Not at the individual level to decide to ‎‎commit suicide.‎ |
| 29. | Witte et al., 2005, U.S.‎[29] | Not using machine learning to predict ‎‎‎‎‎‎‎suicide, suicide ideation, or behavior. ‎‎‎‎‎‎‎The authors assessed the association ‎‎‎‎‎‎‎between suicide and other variables.‎ |
| 30. | Kirlic et al., 2021, U.S.[30] | Not using machine learning to predict ‎‎suicide, suicide ideation, or behavior. ‎‎The authors assessed the association ‎‎between suicide and other variables.‎ |
| 31. | Kuroki, 2015, U.S.[31] | Not using machine learning to predict ‎‎suicide, suicide ideation, or behavior. ‎‎The authors assessed the association ‎‎between suicide and other variables.‎ |
| 32. | Leiva-Murillo, 2013, Spain[32] | Not using machine learning to predict ‎‎suicide, suicide ideation, or behavior. ‎‎The authors assessed the association ‎‎between suicide and other variables.‎ |
| 33. | López et al., 2021, México[33] | Not using machine learning to predict ‎‎‎suicide, suicide ideation, or behavior. ‎‎‎The authors assessed the association ‎‎‎between suicide and other variables.‎ |
| 34. | Lu et al., 2020, China[34] | Not using machine learning to predict ‎‎‎suicide, suicide ideation, or behavior. ‎‎‎The authors assessed the association ‎‎‎between suicide and other variables.‎ |
| 35. | Lucas et al., 2020, U.S.[35] | Not using machine learning to predict ‎‎‎suicide, suicide ideation, or behavior. ‎‎‎The authors assessed the association ‎‎‎between suicide and other variables.‎ |
| 36. | Mukherjee et al., 2021, U.S.[36] | Not at the individual level to decide to ‎‎commit suicide.‎ |
| 37. | Naifeh et al., 2021, U.S.[37] | Not using machine learning to predict ‎‎‎‎suicide, suicide ideation, or behavior. ‎‎‎‎The authors assessed the association ‎‎‎‎between suicide and other variables.‎ |
| 38. | Patel et al., 2020, U.S.[38] | Not using machine learning to predict ‎‎‎‎suicide, suicide ideation, or behavior. ‎‎‎‎The authors assessed the association ‎‎‎‎between suicide and other variables.‎ |
| 39. | Reger et al., 2019, U.S.[39] | Not using machine learning to predict ‎‎‎‎suicide, suicide ideation, or behavior. ‎‎‎‎The authors assessed the association ‎‎‎‎between suicide and other variables.‎ |
| 40. | Reyes-Ruiz et al., 2019, Colombia[40] | Not using machine learning to predict ‎‎‎‎suicide, suicide ideation, or behavior. ‎‎‎‎The authors assessed the association ‎‎‎‎between suicide and other variables.‎ |
| 41. | Ronzitti et al., 2019, U.S.[41] | Not using machine learning to predict ‎‎‎‎suicide, suicide ideation, or behavior. ‎‎‎‎The authors assessed the association ‎‎‎‎between suicide and other variables.‎ |
| 42. | Rostad et al., 2021, U.S.[42] | Not using machine learning to predict ‎‎‎‎suicide, suicide ideation, or behavior. ‎‎‎‎The authors assessed the association ‎‎‎‎between suicide and other variables.‎ |
| 43. | Sewall et al., 2021, U.S.[43] | Not using machine learning to predict ‎‎‎‎suicide, suicide ideation, or behavior. ‎‎‎‎The authors assessed the association ‎‎‎‎between suicide and other variables.‎ |
| 44. | Shen et al., 2022, Taiwan[44] | Not at the individual level to decide to ‎commit suicide.‎ |
| 45. | Sil et al., 2021, India[45] | Not using machine learning to predict ‎‎‎‎‎suicide, suicide ideation, or behavior. ‎‎‎‎‎The authors assessed the association ‎‎‎‎‎between suicide and other variables.‎ |
| 46. | Stuke et al., 2020, 34 countries[46] | Not at the individual level to decide to ‎‎commit suicide.‎ |
| 47. | Tan et al., 2021, China[47] | Not using machine learning to predict ‎‎‎‎‎suicide, suicide ideation, or behavior. ‎‎‎‎‎The authors assessed the association ‎‎‎‎‎between suicide and other variables.‎ |
| 48. | Tasmim et al., 2020, Canada[48] | This article was a letter to the editor |
| 49. | Tran et al., 2015, Australia[49] | Not using machine learning to predict ‎‎‎‎‎‎suicide, suicide ideation, or behavior. ‎‎‎‎‎‎The authors assessed the association ‎‎‎‎‎‎between suicide and other variables.‎ |
| 50. | Tzeng et al., 2006, U.S.[50] | This article was a commentary |
| 51. | Schaik et al., 2019, UK[51] | Not using machine learning to predict ‎‎‎‎‎‎suicide, suicide ideation, or behavior. ‎‎‎‎‎‎The authors assessed the association ‎‎‎‎‎‎between suicide and other variables.‎ |
| 52. | Venek et al., 2017, U.S.[52] | Not using administrative data |
| 53. | Yang et al., 2020, U.S.‎[53] | Not using machine learning to predict ‎‎‎‎‎‎suicide, suicide ideation, or behavior. ‎‎‎‎‎‎The authors assessed the association ‎‎‎‎‎‎between suicide and other variables.‎ |
| 54. | Yang et al., 2020, China[54] | Not using machine learning to predict ‎‎‎‎‎‎suicide, suicide ideation, or behavior. ‎‎‎‎‎‎The authors assessed the association ‎‎‎‎‎‎between suicide and other variables.‎ |
| 55. | Yoon et al., 2021, South Korea[55] | Not using machine learning to predict ‎‎‎‎‎‎suicide, suicide ideation, or behavior. ‎‎‎‎‎‎The authors assessed the association ‎‎‎‎‎‎between suicide and other variables.‎ |
| 56. | Zaborskis et al., 2019,‎ Israel,  Lithuania, and Luxembourg[56] | Not using machine learning to predict ‎‎‎‎‎‎suicide, suicide ideation, or behavior. ‎‎‎‎‎‎The authors assessed the association ‎‎‎‎‎‎between suicide and other variables.‎ |
| 57. | Colic et al., 2022, Canada[57] | This study mixed self-harm with suicide |
| 58. | McCarthy et al., 2015, U.S.‎[58] | Not using machine learning to predict ‎‎‎‎‎‎suicide, suicide ideation, or behavior. ‎‎‎‎‎‎The authors assessed the association ‎‎‎‎‎‎between suicide and other variables.‎ |
| 59. | Mohr et al., 2017, U.S.‎[59] | This article was a review article |
| 60. | Schoenbaum et al., 2014, U.S.‎[60] | Not using machine learning to predict ‎‎‎‎‎‎suicide, suicide ideation, or behavior. ‎‎‎‎‎‎The authors assessed the association ‎‎‎‎‎‎between suicide and other variables.‎ |
| 61. | Tucker et al., 2019, U.S.‎[61] | This article is about ethical considerations of predictive models. |
| 62. | Värnik, et al., 2012, ‎ WHO world database[62] | Not at the individual level to decide to ‎‎‎commit suicide.‎ |
| 63. | Baca-García, et al., 2006, Spain[63] | Not at the individual level to decide to ‎‎‎‎commit suicide.‎ |
| 64. | Barak-Corren‎, et al., 2020, U.S.[64] | Validating a suicide risk tool |
| 65. | Althoff, 2020, U.S.[65] | Full text was not available. |
| 66. | Aupperle et al., 2019, U.S.[66] | Full text was not available. |
| 67. | Alici et al., 2021, Turkey ‎[67] | Full text was not available.‎ |
| 68. | Garza et al., ‎2020, U.S.‎[68] | Full text was not available.‎ |
| 69. | Lewin et al., ‎2019, Australia[69] | Full text was not available.‎ |
| 70. | Kim et al., 2019, U.S.[70] | Full text was not available. |
| 71. | Fradera et al., 2019, Spain[71] | Full text was not available. |
| 72. | Choo et al., 2022, U.S.[72] | Full text was not available. |
| 73. | Cardoso et al., 2021, Brazil [73] | Full text was not available. |
| 74. | Visoki et al., 2021, U.S.‎[74] | Full text was not available.‎ |

1. Alarcao AC, Agnolo CMD, Vissoci JR, Carvalho ECA, Staton CA, de Andrade L, et al. Suicide mortality among youth in southern Brazil: a spatiotemporal evaluation of socioeconomic vulnerability. BRAZILIAN JOURNAL OF PSYCHIATRY 2020 JAN-FEB;42(1):46-53. [PMID: WOS:000512294000010] [doi: 10.1590/1516-4446-2018-0352]

2. Choo CC, Chew PKH, Ho RC. Controlling Noncommunicable Diseases in Transitional Economies: Mental Illness in Suicide Attempters in Singapore—An Exploratory Analysis. BioMed Research International 2019:1-8. [PMID: 134122674. Language: English. Entry Date: 20190118. Revision Date: 20190712. Publication Type: Article] [doi: 10.1155/2019/4652846]

3. Yin XL, Ma D, Zhu KJ, Li DY. Identifying intentional injuries among children and adolescents based on Machine Learning. PLOS ONE 2021 JAN 20;16(1). [PMID: WOS:000639428800058] [doi: 10.1371/journal.pone.0245437]

4. Allen NB, Nelson BW, Brent D, Auerbach RP. Short-term prediction of suicidal thoughts and behaviors in adolescents: Can recent developments in technology and computational science provide a breakthrough? Journal of affective disorders 2019;250:163-9. [doi: 10.1016/j.jad.2019.03.044]

5. Batterham PJ, Christensen H. Longitudinal risk profiling for suicidal thoughts and behaviours in a community cohort using decision trees. J Affect Disord 2012 Dec 15;142(1-3):306-14. [PMID: 22840465] [doi: 10.1016/j.jad.2012.05.021]

6. Bayramli I, Castro V, Barak-Corren Y, Madsen EM, Nock MK, Smoller JW, et al. Temporally informed random forests for suicide risk prediction. Journal of the American Medical Informatics Association 2022;29(1):62-71. [PMID: 154359992. Language: English. Entry Date: 20220318. Revision Date: 20220318. Publication Type: journal article] [doi: 10.1093/jamia/ocab225]

7. Bossarte RM, Kennedy CJ, Luedtke A, Nock MK, Smoller JW, Stokes C, et al. Invited Commentary: New Directions in Machine Learning Analyses of Administrative Data to Prevent Suicide-Related Behaviors Comment. AMERICAN JOURNAL OF EPIDEMIOLOGY 2021 DEC;190(12):2528-33. [PMID: WOS:000743140600005] [doi: 10.1093/aje/kwab111]

8. Brown LA, Benhamou K, May AM, Mu WT, Berk R. Machine Learning Algorithms in Suicide Prevention: Clinician Interpretations as Barriers to Implementation. JOURNAL OF CLINICAL PSYCHIATRY 2020 MAY-JUN;81(3). [PMID: WOS:000616422300007] [doi: 10.4088/JCP.19m12970]

9. Zubizarreta JR, Umhau JC, Deuster PA, Brenner LA, King AJ, Petukhova MV, et al. Evaluating the heterogeneous effect of a modifiable risk factor on suicide: The case of vitamin D deficiency. Int J Methods Psychiatr Res 2022 Mar;31(1):e1897. [PMID: 34739164] [doi: 10.1002/mpr.1897]

10. Chekroud AM. Anticipating Suicide Will Be Hard, But This Is Progress. American Journal of Psychiatry 2018;175(10):921-2. [PMID: 132097689. Language: English. Entry Date: 20190825. Revision Date: 20210513. Publication Type: editorial] [doi: 10.1176/appi.ajp.2018.18060714]

11. Cho YJ. Are Korean children free from suicide? Risk and protective factors within a transactional-ecological perspective. J Child Health Care 2020 Sep;24(3):473-85. [PMID: 31510783] [doi: 10.1177/1367493519874969]

12. Choo C, Diederich J, Song I, Ho R. Cluster analysis reveals risk factors for repeated suicide attempts in a multi-ethnic Asian population. Asian J Psychiatr 2014 Apr;8:38-42. [PMID: 24655624] [doi: 10.1016/j.ajp.2013.10.001]

13. Modai I, Kuperman J, Goldberg I, Goldish M, Mendel S, Modai I, et al. Fuzzy logic detection of medically serious suicide attempt records in major psychiatric disorders. Journal of Nervous & Mental Disease 2004;192(10):708-10. [PMID: 106545388. Language: English. Entry Date: 20051202. Revision Date: 20200708. Publication Type: journal article] [doi: 10.1097/01.nmd.0000142020.20038.dd]

14. Pestian JP, Sorter M, Connolly B, Bretonnel Cohen K, McCullumsmith C, Gee JT, et al. A Machine Learning Approach to Identifying the Thought Markers of Suicidal Subjects: A Prospective Multicenter Trial. Suicide Life Threat Behav 2017 Feb;47(1):112-21. [PMID: 27813129] [doi: 10.1111/sltb.12312]

15. Connolly B, Cohen KB, Santel D, Bayram U, Pestian J. A nonparametric Bayesian method of translating machine learning scores to probabilities in clinical decision support. BMC Bioinformatics 2017 Aug 7;18(1):361. [PMID: 28784111] [doi: 10.1186/s12859-017-1736-3]

16. Desjardins I, Cats-Baril W, Maruti S, Freeman K, Althoff R. Suicide Risk Assessment in Hospitals: An Expert System-Based Triage Tool. J Clin Psychiatry 2016 Jul;77(7):e874-82. [PMID: 27314465] [doi: 10.4088/JCP.15m09881]

17. Edgcomb JB, Shaddox T, Hellemann G, Brooks JO. Predicting suicidal behavior and self-harm after general hospitalization of adults with serious mental illness. Journal of Psychiatric Research 2020:No-Specified. [doi: <https://dx.doi.org/10.1016/j.jpsychires.2020.10.024>]

18. Edgcomb JB, Thiruvalluru R, Pathak J, Brooks Iii JO. Machine Learning to Differentiate Risk of Suicide Attempt and Self-harm After General Medical Hospitalization of Women With Mental Illness. Medical Care 2021;59:S58-S64. [PMID: 149459637. Language: English. Entry Date: In Process. Revision Date: 20210401. Publication Type: Article. Supplement Title: 2021 Supplement. Journal Subset: Biomedical] [doi: 10.1097/mlr.0000000000001467]

19. Mezuk B, Ko TM, Kalesnikava VA, Jurgens D. Suicide Among Older Adults Living in or Transitioning to Residential Long-term Care, 2003 to 2015. JAMA network open 2019;2(6):e195627-e. [PMID: 31199445] [doi: 10.1001/jamanetworkopen.2019.5627]

20. García-Martín Á F, Santiago-Sáez A, Labajo-González E, Albarrán-Juan ME, Olivares-Pardo E, Perea-Pérez B. [Utility of the classification analysis in the typology of the the suicide attempt in a hospital population.]. Rev Esp Salud Publica 2020 Nov 20;94. [PMID: 33215612]

21. Gradus JL, Lash TL, Rosellini AJ, Galatzer-Levy I, Street AE, Jiang T, et al. Gradus et al. Respond to "Machine Learning and Suicide Prevention: New Directions"...Bossarte RM, Kennedy CJ, Luedtke A, et al. Invited commentary: new directions in machine learning analyses of administrative data to prevent suicide-related behaviors. Am J Epidemiol. 2021;190(12):2528–2533. American Journal of Epidemiology 2021;190(12):2534-5. [PMID: 153984548. Language: English. Entry Date: 20211215. Revision Date: 20211215. Publication Type: Article] [doi: 10.1093/aje/kwab113]

22. Hwang LC, Dada O, Adanty C, Fatemi AB, Graff A, Strauss J, et al. Independent effect of childhood trauma and recent stress as predictors for current suicidal ideation in schizophrenia. Gen Hosp Psychiatry 2020 May-Jun;64:115-6. [PMID: 31744635] [doi: 10.1016/j.genhosppsych.2019.09.003]

23. Ibala R-M, Seff I, Stark L. Attitudinal Acceptance of Intimate Partner Violence and Mental Health Outcomes for Female Survivors in Sub-Saharan Africa. International journal of environmental research and public health 2021;18(10). [doi: <https://dx.doi.org/10.3390/ijerph18105099>]

24. Ilgen MA, Downing K, Zivin K, Hoggatt KJ, Kim HM, Ganoczy D, et al. Exploratory data mining analysis identifying subgroups of patients with depression who are at high risk for suicide. J Clin Psychiatry 2009 Nov;70(11):1495-500. [PMID: 20031094] [doi: 10.4088/JCP.08m04795]

25. Jang S-Y, Yang D-S, Cha Y-H, Yoo H-J, Kim K-J, Choy W-S. Suicide in Elderly Patients with Hip Fracture: A South Korean Nationwide Cohort Study. The Journal of bone and joint surgery American volume 2020;102(12):1059-65. [doi: <https://dx.doi.org/10.2106/JBJS.19.01436>]

26. Jiang T. Suicide and non-fatal suicide attempts among persons with depression in the population of Denmark. Dissertation Abstracts International: Section B: The Sciences and Engineering 2021;82(12-B):No-Specified.

27. Kan SK, Chen NN, Zhang YL. Predicting the risk of suicide attempt in a depressed population: Development and assessment of an efficient predictive nomogram. Psychiatry Res 2022 Apr;310:114436. [PMID: 35190339] [doi: 10.1016/j.psychres.2022.114436]

28. Kang HK, Bullman TA, Smolenski DJ, Skopp NA, Gahm GA, Reger MA. Suicide risk among 1.3 million veterans who were on active duty during the Iraq and Afghanistan wars. Ann Epidemiol 2015 Feb;25(2):96-100. [PMID: 25533155] [doi: 10.1016/j.annepidem.2014.11.020]

29. Witte TK, Fitzpatrick KK, Joiner TE, Schmidt NB. Variability in suicidal ideation: A better predictor of suicide attempts than intensity or duration of ideation? Journal of affective disorders 2005;88(2):131-6. [doi: 10.1016/j.jad.2005.05.019]

30. Kirlic N, Akeman E, DeVille DC, Yeh H-W, Cosgrove KT, McDermott TJ, et al. A machine learning analysis of risk and protective factors of suicidal thoughts and behaviors in college students. Journal of American college health;ahead-of-print(ahead-of-print):1-10. [doi: 10.1080/07448481.2021.1947841]

31. Kuroki Y. Risk factors for suicidal behaviors among Filipino Americans: a data mining approach. Am J Orthopsychiatry 2015 Jan;85(1):34-42. [PMID: 25110976] [doi: 10.1037/ort0000018]

32. Leiva-Murillo JM, López-Castromán J, Baca-García E. Characterization of suicidal behaviour with self-organizing maps. Comput Math Methods Med 2013;2013:136743. [PMID: 23864904] [doi: 10.1155/2013/136743]

33. Lopez CMR, Rivera MM, Zezzatti AO, Gallegos JCP, Mendoza JEG. Identification of possible suicide cases using a Bayesian Classifier with the database the Emergency Service 911 of Aguascalientes. INTERNATIONAL JOURNAL OF COMBINATORIAL OPTIMIZATION PROBLEMS AND INFORMATICS 2021 JAN-APR;12(1):43-57. [PMID: WOS:000600506700006]

34. Lu L, Jian S, Dong M, Gao J, Zhang T, Chen X, et al. Childhood trauma and suicidal ideation among Chinese university students: the mediating effect of Internet addiction and school bullying victimisation. Epidemiol Psychiatr Sci 2020 Aug 10;29:e152. [PMID: 32772993] [doi: 10.1017/s2045796020000682]

35. Lucas AG, Chang EC, Li M, Chang OD, Yu EA, Hirsch JK. Trauma and Suicide Risk in College Students: Does Lack of Agency, Lack of Pathways, or Both Add to Further Risk? Soc Work 2020 Apr 1;65(2):105-13. [PMID: 32195550] [doi: 10.1093/sw/swaa007]

36. Mukherjee S, Wei ZY. Suicide disparities across metropolitan areas in the US: A comparative assessment of socio-environmental factors using a data-driven predictive approach. PLOS ONE 2021 NOV 24;16(11). [PMID: WOS:000747234800009] [doi: 10.1371/journal.pone.0258824]

37. Naifeh JA, Mash HBH, Stein MB, Vance MC, Aliaga PA, Fullerton CS, et al. Sex Differences in US Army Suicide Attempts During the Wars in Iraq and Afghanistan. Med Care 2021 Feb 1;59(2 Suppl):S42-s50. [PMID: 33438882] [doi: 10.1097/mlr.0000000000001425]

38. Patel RS, Onyeaka H, Youssef NA. Suicidal ideation and attempts in unipolar versus bipolar depression: analysis of 131,740 adolescent inpatients nationwide. Psychiatry Res 2020 Sep;291:113231. [PMID: 32574899] [doi: 10.1016/j.psychres.2020.113231]

39. Reger GM, McClure ML, Ruskin D, Carter SP, Reger MA. Integrating predictive modeling into mental health care: An example in suicide prevention. Psychiatric Services 2019;70(1):71-4. [doi: <http://dx.doi.org/10.1176/appi.ps.201800242>]

40. Reyes-Ruiz L, Granadillo EJLH, Alvarado FAC. Method to identify and predict suicide risk profiles of adolescents using techniques of conglomerate analysis and artificial neural network. Archivos Venezolanos de Farmacologia y Terapeutica 2019;38(3):115-20.

41. Ronzitti S, Kraus SW, Decker SE, Ashrafioun L. Clinical characteristics of veterans with gambling disorders seeking pain treatment. Addict Behav 2019 Aug;95:160-5. [PMID: 30925440] [doi: 10.1016/j.addbeh.2019.03.014]

42. Rostad WL, Basile KC, Clayton HB. Association Among Television and Computer/Video Game Use, Victimization, and Suicide Risk Among U.S. High School Students. J Interpers Violence 2021 Mar;36(5-6):2282-305. [PMID: 29502506] [doi: 10.1177/0886260518760020]

43. Sewall CJR, Girard JM, Merranko J, Hafeman D, Goldstein BI, Strober M, et al. A Bayesian multilevel analysis of the longitudinal associations between relationship quality and suicidal ideation and attempts among youth with bipolar disorder. JOURNAL OF CHILD PSYCHOLOGY AND PSYCHIATRY 2021 JUL;62(7):905-15. [PMID: WOS:000583509400001] [doi: 10.1111/jcpp.13343]

44. Shen Y-S, Lung S-CC, Cui S. Exploring multiple pathways and mediation effects of urban environmental factors for suicide prevention. Environmental pollution (Barking, Essex : 1987) 2022;294:118642. [doi: <https://dx.doi.org/10.1016/j.envpol.2021.118642>]

45. Sil A, Dhillon P, Mog M. Factors affecting External causes of deaths among adults (15-59 years) in South Indian states: A study using Bayesian model on National Family Health Survey-4 (2015-16) data. Clinical Epidemiology and Global Health 2021;11:100796. [doi: <http://dx.doi.org/10.1016/j.cegh.2021.100796>]

46. Stuke H, Heinz A, Bermpohl F. Acceptance towards lgb persons is an independent protective factor against suicide on a country level. Sexuality Research & Social Policy: A Journal of the NSRC 2020:No-Specified. [doi: <https://dx.doi.org/10.1007/s13178-020-00477-3>]

47. Tan L, Xia T. Predictors on workplace suicidal ideation: From a social interaction perspective. Current Psychology: A Journal for Diverse Perspectives on Diverse Psychological Issues 2021:No-Specified. [doi: <https://dx.doi.org/10.1007/s12144-021-01928-9>]

48. Tasmim S, Dada O, Wang KZ, Bani-Fatemi A, Strauss J, Adanty C, et al. Early-life stressful events and suicide attempt in schizophrenia: Machine learning models. Elsevier B.V.; 2020. p. 329-31.

49. Tran T, Nguyen TD, Phung D, Venkatesh S. Learning vector representation of medical objects via EMR-driven nonnegative restricted Boltzmann machines (eNRBM). J Biomed Inform 2015 Apr;54:96-105. [PMID: 25661261] [doi: 10.1016/j.jbi.2015.01.012]

50. Tzeng HM. Forecasting: Adopting the methodology of support vector machines to nursing research. Worldviews Evid Based Nurs 2006;3(3):124-8. [PMID: 16965314] [doi: 10.1111/j.1741-6787.2006.00062.x]

51. van Schaik P, Peng YH, Ojelabi A, Ling J. Explainable statistical learning in public health for policy development: the case of real-world suicide data. BMC MEDICAL RESEARCH METHODOLOGY 2019 JUL 17;19. [PMID: WOS:000475928300001] [doi: 10.1186/s12874-019-0796-7]

52. Venek V, Scherer S, Morency LP, Rizzo A, Pestian J. Adolescent Suicidal Risk Assessment in Clinician-Patient Interaction. IEEE TRANSACTIONS ON AFFECTIVE COMPUTING 2017 APR-JUN;8(2):204-15. [PMID: WOS:000402709900006] [doi: 10.1109/TAFFC.2016.2518665]

53. Yang J, Liu Q, Zhao F, Feng X, Kaaya RE, Lyu J. Incidence of and sociological risk factors for suicide death in patients with leukemia: A population-based study. J Int Med Res 2020 May;48(5):300060520922463. [PMID: 32383398] [doi: 10.1177/0300060520922463]

54. Yang XY, Wang SS, Eklund L. Reacting to social discrimination? Men's individual and social risk behaviors in the context of a male marriage squeeze in rural China. SOCIAL SCIENCE & MEDICINE 2020 FEB;246. [PMID: WOS:000515194800013] [doi: 10.1016/j.socscimed.2019.112729]

55. Yoon CG, Jung J, Yoon JH, Lee D, Jeon H, Lee SY. How Is the Suicide Ideation in the Korean Armed Forces Affected by Mental Illness, Traumatic Events, and Social Support? J Korean Med Sci 2021 Apr 19;36(15):e96. [PMID: 33876585] [doi: 10.3346/jkms.2021.36.e96]

56. Zaborskis A, Ilionsky G, Tesler R, Heinz A. The association between cyberbullying, school bullying, and suicidality among adolescents: Findings from the cross-national study HBSC in Israel, Lithuania, and Luxembourg. Crisis: The Journal of Crisis Intervention and Suicide Prevention 2019 2019

2020-12-07;40(2):100-14. [PMID: 2097214255; 2018-42356-001] [doi: <https://doi.org/10.1027/0227-5910/a000536>]

57. Colic S, He JC, Richardson JD, St Cyr K, Reilly JP, Hasey GM. A machine learning approach to identification of self-harm and suicidal ideation among military and police Veterans. JOURNAL OF MILITARY VETERAN AND FAMILY HEALTH 2022 FEB 1;8(1):56-67. [PMID: WOS:000731838000002] [doi: 10.3138/jmvfh-2021-0035]

58. McCarthy JF, Bossarte RM, Katz IR, Thompson C, Kemp J, Hannemann CM, et al. Predictive Modeling and Concentration of the Risk of Suicide: Implications for Preventive Interventions in the US Department of Veterans Affairs. Am J Public Health 2015 Sep;105(9):1935-42. [PMID: 26066914] [doi: 10.2105/ajph.2015.302737]

59. Mohr DC, Zhang M, Schueller SM. Personal Sensing: Understanding Mental Health Using Ubiquitous Sensors and Machine Learning. Annual review of clinical psychology 2017;13(1):23-47. [doi: 10.1146/annurev-clinpsy-032816-044949]

60. Schoenbaum M, Kessler RC, Gilman SE, Colpe LJ, Heeringa SG, Stein MB, et al. Predictors of suicide and accident death in the Army Study to Assess Risk and Resilience in Servicemembers (Army STARRS): results from the Army Study to Assess Risk and Resilience in Servicemembers (Army STARRS). JAMA Psychiatry 2014 May;71(5):493-503. [PMID: 24590048] [doi: 10.1001/jamapsychiatry.2013.4417]

61. Tucker RP, Tackett MJ, Glickman D, Reger MA. Ethical and Practical Considerations in the Use of a Predictive Model to Trigger Suicide Prevention Interventions in Healthcare Settings. Suicide Life Threat Behav 2019 Apr;49(2):382-92. [PMID: 29345356] [doi: 10.1111/sltb.12431]

62. Värnik P. Suicide in the world. Int J Environ Res Public Health 2012 Mar;9(3):760-71. [PMID: 22690161] [doi: 10.3390/ijerph9030760]

63. Baca-García E, Perez-Rodriguez MM, Basurte-Villamor I, Saiz-Ruiz J, Leiva-Murillo JM, de Prado-Cumplido M, et al. Using data mining to explore complex clinical decisions: A study of hospitalization after a suicide attempt. J Clin Psychiatry 2006 Jul;67(7):1124-32. [PMID: 16889457] [doi: 10.4088/jcp.v67n0716]

64. Barak-Corren Y, Castro VM, Nock MK, Mandl KD, Madsen EM, Seiger A, et al. Validation of an Electronic Health Record-Based Suicide Risk Prediction Modeling Approach Across Multiple Health Care Systems. JAMA NETWORK OPEN 2020 MAR 25;3(3). [PMID: WOS:000522365600003] [doi: 10.1001/jamanetworkopen.2020.1262]

65. Althoff RR. 8.1 Dysregulation and suicide risk: combining levels of analysis for predicting ‎risk‎. Journal of the American Academy of Child and Adolescent Psychiatry 2020;59(10 Supplement):S277-S8. [doi: <http://dx.doi.org/10.1016/j.jaac.2020.07.590>]

66. Aupperle R, Kuplicki R, DeVille D, Khalsa S, Yeh HW, McDermott T, et al. Psychosocial and Neural Factors Contributing to Suicide Risk in a Large Transdiagnostic Sample. Biological Psychiatry 2019;85(10 Supplement):S116. [doi: <http://dx.doi.org/10.1016/j.biopsych.2019.03.291>]

67. Alici YH, Oztoprak H, Hasanli J, Ozel B, Bagcaz A. Prediction of suicidal behaviour by evaluating personality traids with machine learning techniques. EUROPEAN NEUROPSYCHOPHARMACOLOGY 2021 MAR;44:S34-S5. [PMID: WOS:000624608100047] [doi: 10.1016/j.euroneuro.2021.01.056]

68. de La Garza AG, Blanco C, Olfson M, Wall M. A Data Science Approach to Predicting Future Suicide Attempts With a Large National US Survey. BIOLOGICAL PSYCHIATRY 2020 MAY 1;87(9):S134-S5. [PMID: WOS:000535308200319]

69. Lewin JH, Thompson K, Moncur D, Mancuso S. Predictors of suicide risk in adolescent and young adults (AYA) with cancer. Journal of Clinical Oncology 2019;37(Supplement 15). [doi: <http://dx.doi.org/10.1200/JCO.2019.37.15_suppl.11519>]

70. Kim D, Cogill S, Yang S. An Interpretable Deep Learning Model for the Prevention of Self-Harm and Suicide. ANNALS OF EMERGENCY MEDICINE 2019 OCT;74(4):S6-S. [PMID: WOS:000489265600012] [doi: 10.1016/j.annemergmed.2019.08.014]

71. Fradera M, Morros R, Prat O, Martin-Fumado C, Cardoner N, Palao D, et al. Death due to suicide in barcelona: a case control study to identify main risk factors and opportunities for intervention. European Journal of Clinical Pharmacology 2019;75(Supplement 1):S54. [doi: <http://dx.doi.org/10.1007/s00228-019-02685-2>]

72. Choo TH, Galfalvy H, Stanley B. P694. Effects of Life Events on Suicidal Ideation in EMA Data Using Recurrent Neural Network Prediction. Biological Psychiatry 2022;91(9 Supplement):S371-S2. [doi: <https://dx.doi.org/10.1016/j.biopsych.2022.02.931>]

73. Cardoso T, Ballester P, Moreira FP, Azevedo da Silva R, Mondin TC, Araujo RM, et al. Identifying Nonlinear Patterns of 5-Year Suicide Risk Incidence in Youth: A Gradient Tree Boosting and SHAP Study. Biological Psychiatry 2021;89(9 Supplement):S283. [doi: <https://dx.doi.org/10.1016/j.biopsych.2021.02.705>]

74. Visoki E, Moore T, Gur R, Barzilay R. Prediction of mid adolescent suicide attempt using multidimensional data collected in early adolescence. Neuropsychopharmacology 2021;46:138-9. [doi: <https://dx.doi.org/10.1038/s41386-021-01236-7>]

*Appendix 3*

| PROBAST of included studies | | | | | | | | | | | | | | |
| --- | --- | --- | --- | --- | --- | --- | --- | --- | --- | --- | --- | --- | --- | --- |
| Study | title | ROB | | | | | | | Applicability | | | Overall | |  |
|  |  | Participants | | Predictors | | Outcome | | Analysis | Participants | Predictors | Outcome | ROB | Applicability |  |
|  |  | Dev | Val | Dev | Val | Dev | Val |  |  |  |  |  |  |  |
| Rachel Sayko Adams et al | Sex‐Specific Risk Profiles for Suicide Among Persons with Substance Use Disorders in Denmark | + |  | + |  | + |  | + | + | + | + | + | + |  |
| Neusa Aita Agne et al | Predictors of suicide attempt in patients with obsessive-compulsive disorder: an exploratory study with machine learning analysis | + |  | + |  | ? |  | + | + | + | + | ? | + |  |
| sung Man Bae et al | Prediction by data mining, of suicide attempts in Korean adolescents: a national study | + |  | + |  | + |  | ? | + | + | + | ? | + |  |
| Enrique Baca-‎García et al | Variables associated with familial suicide attempts in a sample of suicide attempters | + |  | + |  | + |  | ? | + | + | + | ? | + |  |
| Pedro L. Ballester | 5-year incidence of suicide-risk in youth: A gradient tree boosting and SHAP study | + |  | + |  | + |  | + | + | + | + | + | + |  |
| Jorge Barros et al | Suicide detection in Chile: proposing a predictive model for suicide risk in a clinical sample of patients with mood disorders | + |  | ? |  | ? |  | + | + | + | + | ? | + |  |
| Samantha L. Bernecker et al | Predicting suicide attempts among soldiers who deny suicidal ideation in the Army Study to Assess Risk and Resilience in Servicemembers (Army STARRS) | + |  | + |  | + |  | + | + | + | + | + | + |  |
| Jennifer M. Buchman-Schmitt et al | Military Suicide Research Consortium common data elements: Bifactor analysis and longitudinal predictive ability of suicidal ideation and suicide attempts within a clinical sample | + |  | + |  | + |  | ? | + | + | + | ? | + |  |
| Taylor A. Burke et al. | Identifying the relative importance of non-suicidal self-injury features in classifying suicidal ideation, plans, and behavior using exploratory data mining | + |  | + |  | + |  | + | + | + | + | + | + |  |
| Qi Chen et al. | Predicting suicide attempt or suicide death following a visit to psychiatric specialty care: A machine learning study using Swedish national registry data | + |  | + |  | + |  | + | + | + | + | + | + |  |
| Seo-Eun Cho et al | Development of a Suicide Prediction Model for the Elderly Using Health Screening Data | + |  | + |  | + |  | + | + | + | + | + | + |  |
| Seo-Eun Cho et al | Prediction of suicide among 372,813 individuals under medical check-up | + |  | + |  | + |  | + | + | + | + | + | + |  |
| Kyu Sung Choi et al | Deep graph neural network-based prediction of acute suicidal ideation in young adults | + | + | + | + | ? | ? | + | + | + | + | ? | + |  |
| Soo Beom Choi et al | Ten-year prediction of suicide death using Cox regression and machine learning in a nationwide retrospective cohort study in South Korea | + |  | + |  | + |  | + | + | + | + | + | + |  |
| Sinisa Colic et al | Using Machine Learning Algorithms to Enhance the Management of Suicide Ideation | + |  | + |  | + |  | + | + | + | + | + | + |  |
| Ronald C. Kessler et al | Using Administrative Data to Predict Suicide After Psychiatric Hospitalization in the Veterans Health Administration System | + |  | + |  | + |  | + | + | + | + | + | + |  |
| E. K. Czyz et al | Predicting short-term suicidal thoughts in adolescents using machine learning: developing decision tools to identify daily level risk after hospitalization | + |  | + |  | ? |  | + | + | + | + | ? | + |  |
| Jessica D. Ribeiro et al | Predicting imminent suicidal thoughts and nonfatal attempts: The role of complexity | + |  | + |  | + |  | + | + | + | + | + | + |  |
| D. Delgado-Gomez et al | Computerized Adaptive Test vs. decision trees: Development of a support decision system to identify suicidal behavior | + |  | + |  | ? |  | + | + | + | + | ? | + |  |
| Marcos DelPozo-Banos et al. | Using Neural Networks with Routine Health Records to Identify Suicide Risk: Feasibility Study | + |  | + |  | ? |  | + | + | + | + | ? | + |  |
| Peihao Fan et al | Prediction of Suicide-Related Events by Analyzing Electronic Medical Records from PTSD Patients with Bipolar Disorder | + |  | + |  | + |  | + | + | + | + | + | + |  |
| Seena Fazel et al | The prediction of suicide in severe mental illness: development and validation of a clinical prediction rule (OxMIS) | + |  | + |  | + |  | + | + | + | + | + | + |  |
| Jaimie L. Gradus et al | Prediction of Sex-Specific Suicide Risk Using Machine Learning and Single-Payer Health Care Registry Data From Denmark | + |  | + |  | + |  | + | + | + | + | + | + |  |
| Jaimie L. Gradus et al | Gender Differences in Machine Learning Models of Trauma and Suicidal Ideation in Veterans of the Iraq and Afghanistan Wars | + |  | + |  | + |  | + | + | + | + | + | + |  |
| Tonelle E. Handley et al | Predictors of suicidal ideation in older people: a decision tree analysis | + |  | + |  | + |  | + | + | + | + | + | + |  |
| Gareth Harman et al | Prediction of suicidal ideation and attempt in 9 and 10 year-old children using transdiagnostic risk features | + |  | + |  | + |  | + | + | + | + | + | + |  |
| Nuwan C. Hettige et al | Classification of suicide attempters in schizophrenia using sociocultural and clinical features: A machine learning approach | + |  | + |  | + |  | + | + | + | + | + | + |  |
| Ryan M. Hill et al | Prospective identification of adolescent suicide ideation using classification tree analysis: Models for community-based screening | + |  | + |  | + |  | + | + | + | + | + | + |  |
| Adam Horvath et al | Predicting suicidal behavior without asking about suicidal ideation: Machine learning and the role of borderline personality disorder criteria | + |  | ? |  | ? |  | - | + | + | + | - | + |  |
| Xieyining Huang et al | The Differences Between Individuals Engaging in Nonsuicidal Self-Injury and Suicide Attempt Are Complex (vs. Complicated or Simple) | + |  | + |  | ? |  | + | + | + | + | ? | + |  |
| Xieyining Huang et all | The Differences Between Suicide Ideators and Suicide Attempters: Simple, Complicated, or Complex? | + |  | + |  | + |  | + | + | + | + | + | + |  |
| Xinlei Ji et al | Highlighting psychological pain avoidance and decision-making bias as key predictors of suicide attempt in major depressive disorder-A novel investigative approach using machine learning | + |  | + |  | + |  | + | + | + | + | + | + |  |
| Joshua T. Jordan et al | Characteristics of a suicide attempt predict who makes another attempt after hospital discharge: A decision-tree investigation | + |  | + |  | + |  | + | + | + | + | + | + |  |
| Pascal Jordan et al | Predicting suicidal ideation in primary care: An approach to identify easily assessable key variables | + |  | + |  | + |  | ? | + | + | + | ? | + |  |
| Jun Su Jung et al | Prediction models for high risk of suicide in Korean adolescents using machine learning techniques | + |  | + |  | + |  | + | + | + | + | + | + |  |
| Ronald C. Kessler et al | Developing a practical suicide risk prediction model for targeting high‐risk patients in the Veterans health Administration | + |  | + |  | + |  | + | + | + | + | + | + |  |
| Ronald C. Kessler et al | Predicting suicides after outpatient mental health visits in the Army Study to Assess Risk and Resilience in Servicemembers (Army STARRS) | + |  | + |  | + |  | + | + | + | + | + | + |  |
| Ronald C. Kessler | Predicting suicides after psychiatric hospitalization in US Army soldiers: the Army Study To Assess Risk and resilience in Servicemembers (Army STARRS)) | + |  | + |  | + |  | + | + | + | + | + | + |  |
| Lee, Yoonju et al | Comparison of the Prediction Model of Adolescents' Suicide Attempt Using Logistic Regression and Decision Tree: Secondary Data Analysis of the 2019 Youth Health Risk Behavior Web-Based Survey | + |  | + |  | + |  | ? | + | + | + | ? | + |  |
| Kyung-Won Kim et al | Classification of Adolescent Psychiatric Patients at High Risk of Suicide Using the Personality Assessment Inventory by Machine Learning | + |  | + |  | + |  | + | + | + | + | + | + |  |
| Sunhae Kim et al | Detecting suicidal risk using MMPI-2 based on machine learning algorithm | + |  | + |  | + |  | ? | + | + | + | ? | + |  |
| Sunhae Kim et al | Which PHQ-9 Items Can Effectively Screen for Suicide? Machine Learning Approaches | + |  | + |  | + |  | + | + | + | + | + | + |  |
| Yusuke Kuroki et al | Recursive partitioning analysis of lifetime suicidal behaviors in Asian Americans | + |  | + |  | + |  | + | + | + | + | + | + |  |
| Jinhee Lee et al | Development of a suicide index model in general adolescents using the South Korea 2012-2016 national representative survey data | + |  | + |  | + |  | + | + | + | + | + | + |  |
| Colin G. Walsh et al | Predicting suicide attempts in adolescents with longitudinal clinical data and machine learning | + |  | + |  | + |  | + | + | + | + | + | + |  |
| Gen-Min Lin et al | Machine Learning Based Suicide Ideation Prediction for Military Personnel | + |  | + |  | ? |  | + | + | + | + | ? | + |  |
| I-Li Lin | Predicting the Risk of Future Multiple Suicide Attempt among First-Time Suicide Attempters: Implications for Suicide Prevention Policy | + |  | + |  | + |  | + | + | + | + | + | + |  |
| Jorge Lopez-Castroman | Distinguishing the relevant features of frequent suicide attempters | + |  | + |  | + |  | + | + | + | + | + | + |  |
| Juncheng Lyu et al | BP neural network prediction model for suicide attempt among Chinese rural residents | + |  | + |  | ? |  | + | + | + | + | ? | + |  |
| Cristiane dos Santos Machado et al | Prediction of suicide attempts in a prospective cohort study with a nationally representative sample of the US population | + |  | + |  | + |  | + | + | + | + | + | + |  |
| Anthony A. Mangino et al | Improving Predictive Classification Models Using Generative Adversarial Networks in the Prediction of Suicide Attempts | + |  | + |  | + |  | ? | + | + | + | + | + |  |
| J. John Mann et al | Classification trees distinguish suicide attempters in major psychiatric disorders: a model of clinical decision making | + |  | + |  | + |  | + | + | + | + | + | + |  |
| G. Marcon et al | Who attempts suicide among medical students? | + |  | + |  | ? |  | + | + | + | + | ? | + |  |
| Lindsey C. McKernan et al | Outpatient Engagement and Predicted Risk of Suicide Attempts in Fibromyalgia | + | + | + | + | + | + | + | + | + | + | + | + |  |
| Ilan Modai et al | Backpropagation and adaptive resonance theory in predicting suicidal risk | + |  | + |  | + |  | + | + | + | + | + | + |  |
| Marcel Miché et al | Prospective prediction of suicide attempts in community adolescents and young adults, using regression methods and machine learning | + |  | + |  | + |  | + | + | + | + | + | + |  |
| Ilan Modai et al | Neural network detection of files of suicidal patients and suicidal profiles | + |  | + |  | + |  | + | + | + | + | + | + |  |
| Susana Morales et al | Acute Mental Discomfort Associated with Suicide Behavior in a Clinical Sample of Patients with Affective Disorders: Ascertaining Critical Variables Using Artificial Intelligence Tools | + |  | + |  | + |  | + | + | + | + | + | + |  |
| Kyoung-Sae Na ey al | The Development of a Suicidal Ideation Predictive Model for Community-Dwelling Elderly Aged >55 Years | + |  | + |  | + |  | + | + | + | + | + | + |  |
| Azam Naghavi et al | Accurate Diagnosis of Suicide Ideation/Behavior Using Robust Ensemble Machine Learning: A University Student Population in the Middle East and North Africa (MENA) Region | + |  | + |  | + |  | + | + | + | + | + | + |  |
| Noratikah Nordin et al | A comparative study of machine learning techniques for suicide attempts predictive model | + |  | + |  | ? |  | + | + | + | + | ? | + |  |
| Bumjo Oh et al | Prediction of Suicidal Ideation among Korean Adults Using Machine Learning: A Cross-Sectional Study | + |  | + |  | + |  | + | + | + | + | + | + |  |
| Jihoon Oh et al | Classification of Suicide Attempts through a Machine Learning Algorithm Based on Multiple Systemic Psychiatric Scales | + |  | + |  | + |  | + | + | + | + | + | + |  |
| Neelang Parghi et al | Assessing the predictive ability of the suicide crisis inventory for near-term suicidal behavior using machine learning approaches | + |  | + |  | + |  | - | + | + | + | - | + |  |
| Ives Cavalcante Passos et al | Identifying a clinical signature of suicidality among patients with mood disorders: A pilot study using a machine learning approach | + |  | + |  | + |  | + | + | + | + | + | + |  |
| Anthony J. Rosellini et al | Using self-report surveys at the beginning of service to develop multi-outcome risk models for new soldiers in the U.S. Army | + |  | + |  | + |  | ? | + | + | + | ? | + |  |
| Anthony J. Rosellininet al | Predeployment predictors of psychiatric disorder-symptoms and interpersonal violence during combat deployment | + |  |  |  | + |  | ? | + | + | + | ? | + |  |
| Seunghyong Ryu et al | Detection of Suicide Attempters among Suicide Ideators Using Machine Learning | + |  | + |  | + |  | + | + | + | + | + | + |  |
| Seunghyong Ryu et al | Use of a Machine Learning Algorithm to Predict Individuals with Suicide Ideation in the General Population | + |  | + |  | + |  | + | + | + | + | + | + |  |
| Michael Sanderson et al | Predicting death by suicide using administrative health care system data: Can recurrent neural network, one-dimensional convolutional neural network, and gradient boosted trees models improve prediction performance? | + |  | + |  | + |  | + | + | + | + | + | + |  |
| Michael Sanderson et al | Predicting death by suicide following an emergency department visit for parasuicide with administrative health care system data and machine learning | + |  | + |  | + |  | + | + | + | + | + | + |  |
| Michael Sanderson et al | Predicting death by suicide using administrative health care system data: Can feedforward neural network models improve upon logistic regression models? | + |  | + |  | + |  | + | + | + | + | + | + |  |
| Jennifer L. Shaw et al | Validating a predictive algorithm for suicide risk with Alaska Native populations | + |  | + |  | + |  | ? | + | + | + | ? | + |  |
| Yanmei Shen et al | Detecting risk of suicide attempts among Chinese medical college students using a machine learning algorithm | + |  | + |  | + |  | + | + | + | + | + | + |  |
| Gregory E. Simon et al | Predicting Suicide Attempts and Suicide Deaths Following Outpatient Visits Using Electronic Health Records | + |  | + |  | + |  | + | + | + | + | + | + |  |
| Gregory E Simon et al | What health records data are required for accurate prediction of suicidal behavior? | + |  | + |  | + |  | + | + | + | + | + | + |  |
| Ian H. Stanley et al | Predicting suicide attempts among US Army soldiers after leaving active duty using information available before leaving active duty: results from the Study to Assess Risk and Resilience in Servicemembers-Longitudinal Study (STARRS-LS) | + |  | + |  | + |  | + | + | + | + | + | + |  |
| Truyen Tran et al | Risk stratification using data from electronic medical records better predicts suicide risks than clinician assessments | + |  | + |  | + |  | + | + | + | + | + | + |  |
| María Tubío-Fungueiriño et al | Viability Study of Machine Learning-Based Prediction of COVID-19 Pandemic Impact in Obsessive-Compulsive Disorder Patients | + |  | + |  | ? |  | ? | + | + | + | ? | + |  |
| Kasper van Mens et al | Predicting future suicidal behaviour in young adults, with different machine learning techniques: A population-based longitudinal study | + |  | + |  | + |  | + | + | + | + | + | + |  |
| Ignacio Peis et ‎al | Deep Sequential Models for Suicidal Ideation From Multiple Source ‎Data | ‎+ |  | ‎+‎ |  | ‎?‎ |  | ‎?‎ | ‎+‎ | ‎+‎ | ‎+‎ | ‎?‎ | ‎+‎ |  |
| Gemma T. Wallace et al | Classification trees identify shared and distinct correlates of nonsuicidal self-injury and suicidal ideation across gender identities in emerging adults | + |  | + |  | + |  | + | + | + | + | + | + |  |
| Colin G.Walsh et al | Prospective Validation of an Electronic Health Record-Based, Real-Time Suicide Risk Model | + |  | + |  | + |  | + | + | + | + | + | + |  |
| Colin G. Walsh et al | Predicting Risk of Suicide Attempts Over Time Through Machine Learning | + |  | + |  | + |  | + | + | + | + | + | + |  |
| Shirley B.Wang et al | A Pilot Study Using Frequent Inpatient Assessments of Suicidal Thinking to Predict Short-Term Postdischarge Suicidal Behavior | + |  | + |  | + |  | + | + | + | + | + | + |  |
| Yan-Xin Wei et al | Prediction of recurrent suicidal behavior among suicide attempters with Cox regression and machine learning: a 10-year prospective cohort study | + |  | + |  | + |  | + | + | + | + | + | + |  |
| Zhiyuan Wei et al | Health-Behaviors Associated With the Growing Risk of Adolescent Suicide Attempts: A Data-Driven Cross-Sectional Study | + |  | + |  | ? |  | ? | + | + | + | ? | + |  |
| Orion Weller et al | Predicting suicidal thoughts and behavior among adolescents using the risk and protective factor framework: A large-scale machine learning approach | + |  | + |  | + |  | ? | + | + | + | ? | + |  |
| Bojan Zalar et al | Suicide and Suicide Attempt Descriptors by Multimethod Approach | + |  | ? |  | ? |  | ? | + | + | + | ? | + |  |
| Le Zheng et al | Development of an early-warning system for high-risk patients for suicide attempt using deep learning and electronic health records | + |  | + |  | + |  | + | + | + | + | + | + |  |
| Kelly L. Zuromski et al | Pre-deployment predictors of suicide attempt during and after combat deployment: Results from the Army Study to Assess Risk and Resilience in Servicemembers | + |  | + |  | + |  | + | + | + | + | + | + |  |
| Hong, Sehoon et al. | Identification of High-risk Groups of Suicide from the Depressed Elderly using Decision Tree Analysis | + |  | + |  | + |  | + | + | + | + | + | + |  |
| Payam Amini et al | Evaluating the High Risk Groups for Suicide: A Comparison of Logistic Regression, Support Vector Machine, Decision Tree and Artificial Neural Network | + |  | ? |  | + |  | + | + | + | + | + | + |  |
| Yuval Barak-Corren et al | Predicting Suicidal Behavior From Longitudinal Electronic Health Records | + |  | + |  | + |  | + | + | + | + | + | + |  |
| Ángel García de la Garza et al | Identification of Suicide Attempt Risk Factors in a National US Survey Using Machine Learning | + |  | + |  | + |  | + | + | + | + | + | + |  |
| Chandan Karmakar et al | Predicting Risk of Suicide Attempt Using History of Physical Illnesses From Electronic Medical Records | + |  | + |  | + |  | + | + | + | + | + | + |  |
| David C. Rozek et al | Using Machine Learning to Predict Suicide Attempts in Military Personnel | + |  | + |  | + |  | + | + | + | + | + | + |  |
| Jorge Barros et al | Recognizing states of psychological vulnerability to suicidal behavior: a Bayesian network of artificial intelligence applied to a clinical sample | + |  | + |  | + |  | + | + | + | + | + | + |  |
| Taylor A. Burke et al | Using machine learning to classify suicide attempt history among youth in medical care settings | + |  | + |  | + |  | + | + | + | + | + | + |  |
| Fenfen Ge et al | Identifying Suicidal Ideation Among Chinese Patients with Major Depressive Disorder: Evidence from a Real-World Hospital-Based Study in China | + |  | ? |  | + |  | + | + | + | + | ? | + |  |
| Emily E. Haroz et al | Reaching Those at Highest Risk for Suicide: Development of a Model Using Machine Learning Methods for use With Native American Communities | + |  | + |  | + |  | + | + | + | + | + | + |  |
| Ryan M. Hill et al | Using Machine Learning to Identify Suicide Risk: A Classification Tree Approach to Prospectively Identify Adolescent Suicide Attempters | + |  | + |  | + |  | + | + | + | + | + | + |  |
| G. Indrawan et al | Smooth support vector machine for suicide-related behaviours prediction | + |  | + |  | + |  | ? | + | + | + | ? | + |  |
| Marie‐Hélène Metzger et al | Use of emergency department electronic medical records for automated epidemiological surveillance of suicide attempts: a French pilot study | + |  | + |  | + |  | + | + | + | + | + | + |  |
| PROBAST = Prediction model Risk Of Bias ASsessment Tool  ROB = risk of bias.  +: Low ROB or low concern in applicability  −: High ROB or high concern in applicability  ?: Unclear ROB or unclear concern in applicability | | | | | | | | | | | | | | |
